# A model of auto immune response

**DOI:** 10.1186/s12865-017-0208-x

**Published:** 2017-06-21

**Authors:** James K. Peterson, Alison M. Kesson, Nicholas J. C. King

**Affiliations:** 1 Department of Biological Sciences, Department of Mathematical Sciences, Clemson University, Martin Hall O-304, BOX 340975, Clemson, 340975 SC USA; 2 Discipline of Child and Adolescent Health and the Marie Bashir Institute for Emerging Infectious Diseases and Biosecurity, University of Sydney, Sydney, NSW Australia; 3 Sydney Children’s Hospital Network, Westmead, Sydney, NSW Australia; 4 Discipline of Pathology, University of Sydney, Sydney, NSW Australia

**Keywords:** Second messenger models, Abstract triggering agent, Signalling agent mediation, Host self damage and death, Oscillation in health levels and remission, Auto immune responses

## Abstract

**Background:**

In this work, we develop a theoretical model of an auto immune response. This is based on modifications of standard second messenger trigger models using both signalling pathways and diffusion and a macro level dynamic systems approximation to the response of a triggering agent such as a virus, bacteria or environmental toxin.

**Results:**

We show that there, in general, will be self damage effects whenever the triggering agent’s effect on the host can be separated into two distinct classes of cell populations.

In each population, the trigger acts differently and this behavior is mediated by the nonlinear interactions between two signalling agents.

**Conclusion:**

If these interactions satisfy certain critical assumptions this will lead to collateral damage. If the initial triggering agent’s action involves any critical host cell population whose loss can lead to serious host health issues, then there is a much increased probability of host death.

Our model also shows that if the nonlinear interaction assumptions are satisfied, there is a reasonable expectation of oscillatory behavior in host health; i.e. periods of remission.

## Background

In [1, 2] we explore a micro level simulation model of a single host’s response to varying levels of West Nile Virus (WNV) infection. In that infection, there is a substantial self damage component and in those papers, we show that this is probably due to the way that the virus infects two cell populations differently. This difference, which involves an larger upregulation of MHC-1 sites on the surface of nondividing infected cells over dividing infected cells, is critical in establishing a self damage or collateral damage response. In [3], we develop a macro level model of the nonlinear interactions between two critical *signalling* agents that mediate the interaction between these two sets of cell populations. In the case of WNV infection, the two signals are the MHC-1 upregulation level of the cell and the free WNV antigen level. This macro model allowed us to predict a host’s health response to varying levels of initial virus dose. Hence, we could begin to understand the oscillations in collateral damage and host health that lead to the survival data we see in WNV infections. The more general musings of [3] will now be extended to the setting of auto immune interactions in general. The derivations here use standard ideas from advanced calculus and differential equations.

## The ***CMN*** model

We assume we have a large population of cells ***T*** which consists of cells which are infected or altered in two distinct ways by a trigger ***V***. based on signals ***I***, ***J*** and ***K***. These two distinct populations of cells will be labeled ***M*** and ***N***. There are also non infected cells, ***H*** and non infected cells which will be removed due to auto immune action which we call ***C***, for collateral damage. We will be using the same approach to studying nonlinear interactions that was used in [3].

We assume the dynamics here are

$$\begin{array}{@{}rcl@{}} \boldsymbol{C}^{\prime}(t) &=& F_{1}(\boldsymbol{C}, \boldsymbol{M}, \boldsymbol{N})\\ \boldsymbol{M}^{\prime}(t) &=& F_{2}(\boldsymbol{C}, \boldsymbol{M}, \boldsymbol{N}))\\ \boldsymbol{N}^{\prime}(t) &=& F_{3}(\boldsymbol{C}, \boldsymbol{M}, \boldsymbol{N})) \end{array} $$

There are then three nonlinear interaction functions *F*_1_,*F*_2_ and *F*_3_ because we know ***C***,***M*** and ***N*** depend on each other’s levels in very complicated ways. Usually, we assume the initial trigger dose ***V***_***0***_ gives rise to some fraction of infected cells and the effect of the trigger will be different in the two cell populations ***M*** and ***N***.

### **Assumption 1**

We assume the number of infected cells is *p*_0_ ***V***_***0***_ which is split into *p*_1_ *p*_0_ ***V***_***0***_ in population ***N*** and *p*_2_ *p*_0_ ***V***_***0***_ in ***M***, where *p*_1_+*p*_2_=1.

For example, a reasonable choice is *p*_1_=0.99 and *p*_2_=0.01. Thus, the total amount of trigger that goes into altered cells is *p*_0_ ***V***_***0***_ and the amount of free trigger is therefore (1−*p*_0_) ***V***_***0***_. Thus, we could expect ***C***_***0***_=0, ***M***_***0***_=*p*_2_ *p*_0_ ***V***_***0***_ and ***N***_***0***_=***M***_***0***_=*p*_1_ *p*_0_ ***V***_***0***_. However, we will explicitly assume we are starting from a point of equilibrium prior to the administration of the viral dose ***V***_***0***_. We could assume there is always some level of collateral damage, ***C***_***0***_ in a host, but we will not do that. We will therefore assume ***C***, ***M*** and ***C*** have achieved these values ***C***_***0***_=0, ***M***_***0***_=0 and ***N***_***0***_=0 right before the moment of alteration by the trigger. Hence, we don’t expect to there to be initial contribution to ***C*** ^′^(0), ***M*** ^′^(0) and ***N*** ^′^(0); i.e. *F*_1_(***C***_***0***_,***M***_***0***_,***N***_***0***_)=0, *F*_2_(***C***_***0***_,***M***_***0***_,***N***_***0***_)=0 and *F*_3_(***C***_***0***_,***M***_***0***_,***N***_***0***_)=0. We are interested in the deviation of ***C***, ***M*** and ***N*** from their optimal values ***C***_***0***_, ***M***_***0***_ and ***N***_***0***_, so let ***c***=***C***−***C***_***0***_, ***m***=***M***−***M***_***0***_ and ***n***=***N***−***N***_***0***_. We can then write ***C***=***C***_***0***_+***c***, ***M***=***M***_***0***_+***m*** and ***N***=***N***_***0***_+***n*** The model can then be rewritten as

$$\begin{array}{@{}rcl@{}} \left(\boldsymbol{C_{0}} + \boldsymbol{c}\right)^{\prime}(t) &=& F_{1}\left(\boldsymbol{C_{0}} + \boldsymbol{c},\boldsymbol{M_{0}} + \boldsymbol{m}, \boldsymbol{N_{0}} + \boldsymbol{n}\right) \\ \left(\boldsymbol{M_{0}} + \boldsymbol{m}\right)^{\prime}(t) &=& F_{2}\left(\left(\boldsymbol{C_{0}} + \boldsymbol{c},\boldsymbol{M_{0}} + \boldsymbol{m}, \boldsymbol{N_{0}} + \boldsymbol{n}\right)\right.\\ \left(\boldsymbol{M_{0}} + \boldsymbol{M}\right)^{\prime}(t) &=& F_{3}\left(\left(\boldsymbol{C_{0}} + \boldsymbol{c},\boldsymbol{M_{0}} + \boldsymbol{m}, \boldsymbol{N_{0}} + \boldsymbol{n}\right)\right. \end{array} $$

or

$$\begin{array}{@{}rcl@{}} \boldsymbol{c}^{\prime}(t) &=& F_{1}(\boldsymbol{C_{0}} + \boldsymbol{c},\boldsymbol{M_{0}} + \boldsymbol{m},,\boldsymbol{N_{0}} + \boldsymbol{n}) \\ \boldsymbol{m}^{\prime}(t) &=& F_{2}((\boldsymbol{C_{0}} + \boldsymbol{c},\boldsymbol{M_{0}} + \boldsymbol{m}, \boldsymbol{N_{0}} + \boldsymbol{n})\\ \boldsymbol{n}^{\prime}(t) &=& F_{3}((\boldsymbol{C_{0}} + \boldsymbol{c},\boldsymbol{M_{0}} + \boldsymbol{m}, \boldsymbol{N_{0}} + \boldsymbol{n}) \end{array} $$

Next, we do a standard tangent plane approximation on the nonlinear dynamics functions *F*_1_,*F*_2_ and *F*_3_ to derive approximation dynamics. The mathematics behind this approximation come from multivariate calculus and can easily be reviewed if required. We find the approximate dynamics are

$$\begin{array}{@{}rcl@{}} \left[\begin{array}{c} \boldsymbol{c}^{\prime}\\ \boldsymbol{m}^{\prime}\\ \boldsymbol{n}^{\prime} \end{array}\right] \approx \left[\begin{array}{ccc} F_{1\boldsymbol{c}}^{o} & F_{1\boldsymbol{m}}^{o} & F_{1\boldsymbol{n}}^{o} \\ F_{2\boldsymbol{c}}^{o} & F_{2\boldsymbol{m}}^{o} & F_{2\boldsymbol{n}}^{o} \\ F_{3\boldsymbol{c}}^{o} & F_{3\boldsymbol{m}}^{o} & F_{3\boldsymbol{n}}^{o} \end{array}\right] \left[\begin{array}{c} \boldsymbol{c}\\ \boldsymbol{m}\\ \boldsymbol{n} \end{array}\right] \end{array} $$

where we now use a standard subscript scheme to indicate the partials. Now let’s add the signals IFN- *γ* (***I***), ***J*** and ***K*** to the mix.

## The CDN IJK model

We can think each variable ***C***, ***M*** and ***N*** as depending on ***I***, ***J*** and ***K***. Thus, we have

$$\begin{array}{@{}rcl@{}} F_{1}(\boldsymbol{C}(\boldsymbol{I,J,K}), \boldsymbol{M}(\boldsymbol{I,J,K}), \boldsymbol{N}(\boldsymbol{I,J,K})) &=& H_{1}(\boldsymbol{I,J,K})\\ F_{2}(\boldsymbol{C}(\boldsymbol{I,J,K}), \boldsymbol{M}(\boldsymbol{I,J,K}), \boldsymbol{N}(\boldsymbol{I,J,K})) &=& H_{2}(\boldsymbol{I,J,K})\\ F_{3}(\boldsymbol{C}(\boldsymbol{I,J,K}), \boldsymbol{M}(\boldsymbol{I,J,K}), \boldsymbol{N}(\boldsymbol{I,J,K})) &=& H_{3}(\boldsymbol{I,J,K}) \end{array} $$

We assume the dynamics here are then

$$\begin{array}{@{}rcl@{}} \boldsymbol{C}^{\prime} &=& H_{1}(\boldsymbol{I,J,K}) \\ \boldsymbol{M}^{\prime}&=& H_{2}(\boldsymbol{I,J,K}) \\ \boldsymbol{N}^{\prime}&=& H_{3}(\boldsymbol{I,J,K}) \end{array} $$

As before assume ***C***, ***M*** and ***C*** have achieved the same optimal values ***C***_***0***_=0, ***M***_***0***_=0 and ***N***_***0***_=0 prior to the moment of infection with trigger dose ***V***_***0***_. These correspond to the starting values prior to exposure to the trigger of ***I***_***0***_, ***J***_***0***_ and ***K***_***0***_. Initially, we don’t expect IFN- *γ* signals so ***I***_***0***_=0. Eventually, we do expect some level of change in ***J*** and ***K*** due to this initial dose and we will assume this change to be proportional to the level of the dose ***V***_***0***_ applied; that is, we will assume this is a simple scaling factor, i.e. ***J***_***0***_=*q*_1_ ***V***_***0***_ for some suitable parameter *q*_1_. Also, once the trigger has been applied dose, we would expect some fraction of it to remain free which will be modeled as ***K***_***0***_=(1−*p*_0_)***V***_***0***_. But now, we think of all the initial values as zero; i.e. ***I***_***0***_=0, ***J***_***0***_=0 and ***K***_***0***_=0. We still don’t expect to have any contribution to ***C*** ^′^(0), ***M*** ^′^(0) and ***N*** ^′^(0); i.e. *H*_1_(***I***_***0***_,***J***_***0***_,***K***_***0***_)=0, *H*_2_(***I***_***0***_,***J***_***0***_,***K***_***0***_)=0 and *H*_3_(***I***_***0***_,***J***_***0***_,***K***_***0***_)=0. We are interested in the deviation of ***C***, ***M*** and ***N*** from their optimal values ***C***_***0***_, ***M***_***0***_ and ***N***_***0***_ due to the changes ***i***, ***j*** and ***k*** from the base ***I***, ***J*** and ***K*** values. So as usual, let ***c***=***C***−***C***_***0***_, ***m***=***M***−***M***_***0***_ and ***n***=***N***−***N***_***0***_. We can then write ***C***=***C***_***0***_+***c***, ***M***=***M***_***0***_+***m*** and ***N***=***N***_***0***_+***n*** The model can then be rewritten as

$$\begin{array}{@{}rcl@{}} \left(\boldsymbol{C_{0}} + \boldsymbol{c}\right)^{\prime}(t) &=& H_{1}\left(\boldsymbol{I_{0}} + \boldsymbol{i},\boldsymbol{J_{0}} + \boldsymbol{j}, \boldsymbol{K_{0}} + \boldsymbol{k}\right) \\ \left(\boldsymbol{M_{0}} + \boldsymbol{m}\right)^{\prime}(t) &=& H_{2}\left(\boldsymbol{I_{0}} + \boldsymbol{i},\boldsymbol{J_{0}} + \boldsymbol{j}, \boldsymbol{K_{0}} + \boldsymbol{k}\right)\\ \left(\boldsymbol{N_{0}} + \boldsymbol{n}\right)^{\prime}(t) &=& H_{3}\left(\boldsymbol{I_{0}} + \boldsymbol{i},\boldsymbol{J_{0}} + \boldsymbol{j}, \boldsymbol{K_{0}} + \boldsymbol{k}\right) \end{array} $$

which, as usual, implies

$$\begin{array}{@{}rcl@{}} \boldsymbol{c}^{\prime}(t) &=& H_{1}\left(\boldsymbol{I_{0}} + \boldsymbol{i},\boldsymbol{J_{0}} + \boldsymbol{j}, \boldsymbol{K_{0}} + \boldsymbol{k}\right) \\ \boldsymbol{m}^{\prime}(t) &=& H_{2}\left(\boldsymbol{I_{0}} + \boldsymbol{i},\boldsymbol{J_{0}} + \boldsymbol{j}, \boldsymbol{K_{0}} + \boldsymbol{k}\right)\\ \boldsymbol{n}^{\prime}(t) &=& H_{3}\left(\boldsymbol{I_{0}} + \boldsymbol{i},\boldsymbol{J_{0}} + \boldsymbol{j}, \boldsymbol{K_{0}} + \boldsymbol{k}\right) \end{array} $$

Next, we again perform a tangent plane approximation on the nonlinear dynamics functions *H*_1_,*H*_2_ and *H*_3_ the details of which are not shown. We find the nonlinear dynamics approximation is then

$$\begin{array}{@{}rcl@{}} \left[\begin{array}{c} \boldsymbol{c}^{\prime}\\ \boldsymbol{m}^{\prime}\\ \boldsymbol{n}^{\prime} \end{array}\right] \approx \left[\begin{array}{ccc} H_{1\boldsymbol{i}}^{o} & H_{1\boldsymbol{j}}^{o} & H_{1\boldsymbol{k}}^{o} \\ H_{2\boldsymbol{i}}^{o} & H_{2\boldsymbol{j}}^{o} & H_{2\boldsymbol{k}}^{o} \\ H_{3\boldsymbol{i}}^{o} & H_{3\boldsymbol{j}}^{o} & H_{3\boldsymbol{k}}^{o} \end{array}\right] \left[\begin{array}{c} \boldsymbol{i}\\ \boldsymbol{J}\\ \boldsymbol{K} \end{array}\right] \end{array} $$

where we now use a standard subscript scheme to indicate the partials. If we hold everything constant except ***i*** which we increase to ***i***+*δ* ***i***, what happens? Increasing the IFN- *γ* level should increase collateral damage. Hence, $H_{1\boldsymbol {i}}^{o} = +$. What about the other two cell populations, ***M*** and ***N***?

### **Assumption 2**

We assume this increase in ***i*** has no effect. Hence, $H_{2\boldsymbol {i}}^{o} = H_{3\boldsymbol {i}}^{o} = 0$.

This assumption is similar to the one we made in [3] and we think it is important to the eventual auto immune response. Thus, the coefficient matrix above which we call ***Ψ*** so far looks like

$$\begin{array}{@{}rcl@{}} \boldsymbol{\Psi} &=& \left[\begin{array}{ccc} + & H_{1\boldsymbol{j}}^{o} & H_{1\boldsymbol{k}}^{o} \\ 0 & H_{2\boldsymbol{j}}^{o} & H_{2\boldsymbol{k}}^{o} \\ 0 & H_{3\boldsymbol{j}}^{o} & H_{3\boldsymbol{k}}^{o} \end{array}\right] \end{array} $$

Now hold everything constant except ***j*** and increase ***j*** to ***j***+*δ* ***j***. What happens?

### **Assumption 3**

What happens will depend on what the signals ***J*** and ***K*** are. We can’t argue much yet. However, we suspect the critical assumption to make is the increase in ***j*** causes ***M*** and ***N*** to decrease. Hence, we assume $H_{2\boldsymbol {j}}^{o} = -$ and $H_{3\boldsymbol {j}}^{o} = -$ for each type of infected cell population.

Thus, the coefficient matrix looks like

$$\begin{array}{@{}rcl@{}} \boldsymbol{\Psi} &=& \left[\begin{array}{ccc} + & + & H_{1\boldsymbol{i}}^{o} \\ 0 & - & H_{2\boldsymbol{K}}^{o} \\ 0 & - & H_{3\boldsymbol{K}}^{o} \end{array}\right] \end{array} $$

Next hold everything constant except the signal level ***k*** and increase ***k*** to ***k***+*δ* ***k***. What happens?

### **Assumption 4**

We assume our choice of signals gives $H_{2\boldsymbol {k}}^{o} = +$ and $H_{3\boldsymbol {k}}^{o} = -$.

Thus, the coefficient matrix now looks like

$$\begin{array}{@{}rcl@{}} \boldsymbol{\Psi} &=& \left[\begin{array}{ccc} + & + & + \\ 0 & - & +\\ 0 & - & - \end{array}\right] \end{array} $$

Or letting $H_{2\boldsymbol {j}}^{o} = -a, H_{3\boldsymbol {j}}^{o} = -b, H_{2\boldsymbol {k}}^{o} = c$, $H_{3\boldsymbol {k}}^{o} = d$, we have the coefficient matrix now looks like

$$\begin{array}{@{}rcl@{}} \boldsymbol{\Psi} &=& \left[\begin{array}{ccc} + & + & + \\ 0 & - a & c\\ 0 & - b & -d \end{array}\right] \end{array} $$

Thus, we have the changes

$$\begin{array}{@{}rcl@{}} \boldsymbol{c}^{\prime} &=& H_{1\boldsymbol{i}}^{o} \: \boldsymbol{i} + H_{1\boldsymbol{j}}^{o} \: \boldsymbol{j} + H_{1\boldsymbol{k}}^{o} \: \boldsymbol{k}\\ \boldsymbol{m}^{\prime} &=& H_{2\boldsymbol{j}}^{o} \: \boldsymbol{j} + H_{2\boldsymbol{k}}^{o} \: \boldsymbol{k}\\ \boldsymbol{n}^{\prime} &=& H_{3\boldsymbol{j}}^{o} \: \boldsymbol{J} + H_{3\boldsymbol{k}}^{o} \: \boldsymbol{k} \end{array} $$

Now we need to estimate ***i***,***j*** and ***k***.

## The IJK model

The amount of ***I***,***J*** and ***K*** depend on the initial amount of trigger applied when in the equilibrium state; i.e. this is the amount that causes the initial infection. This is ***V***_***o***_. We assume the dynamics here are

$$\begin{array}{@{}rcl@{}} \boldsymbol{I}^{\prime} &=& G_{1}(\boldsymbol{I,J,K}) \\ \boldsymbol{J}^{\prime}&=& G_{2}(\boldsymbol{I,J,K}) \\ \boldsymbol{K}^{\prime}&=& G_{3}(\boldsymbol{I,J,K}) \end{array} $$

In the model of “The CDN IJK model” section, we assumed ***C***,***M*** and ***N*** depended on the perturbations of ***I***,***J*** and ***K*** from a zero state. Now, we want to model the ***I***,***J*** and ***K*** deviations from a base state ***I***_***0***_,***J***_***0***_ and ***K***_***0***_ which is not zero. As previously discussed, we expect ***K***_***0***_=(1−*p*_0_) ***V***_***0***_, the initial IFN- *γ* level ***I***_***0***_=0 and the initial upregulation level ***J***_***0***_=*q*_1_ ***V***_***0***_. Let the deviations from these equilibrium values be given by ***i***=***I***−***I***_***0***_, ***J***=***J***−***J***_***0***_ and ***k***=***K***−***K***_***0***_. The model can then be rewritten as

$$\begin{array}{@{}rcl@{}} \left(\boldsymbol{I_{0}} + \boldsymbol{i}\right)^{\prime}(t) &=& G_{1}\left(\boldsymbol{i}+\boldsymbol{I_{0}},\boldsymbol{j}+\boldsymbol{J_{0}}, \boldsymbol{k}+\boldsymbol{K_{0}}\right) \\ \left(\boldsymbol{J_{0}} + \boldsymbol{j}\right)^{\prime}(t) &=& G_{2}\left(\boldsymbol{i}+\boldsymbol{I_{0}},\boldsymbol{j}+\boldsymbol{J_{0}}, \boldsymbol{k}+\boldsymbol{K_{0}}\right) \\ \left(\boldsymbol{K_{0}} + \boldsymbol{k}\right)^{\prime}(t) &=& G_{3}\left(\boldsymbol{i}+\boldsymbol{I_{0}},\boldsymbol{j}+\boldsymbol{J_{0}}, \boldsymbol{k}+\boldsymbol{K_{0}}\right) \end{array} $$

or

$$\begin{array}{@{}rcl@{}} \boldsymbol{i}^{\prime}(t) &=& G_{1}\left(\boldsymbol{i}+\boldsymbol{I_{0}},\boldsymbol{j}+\boldsymbol{J_{0}}, \boldsymbol{k}+\boldsymbol{K_{0}}\right) \\ \boldsymbol{j}^{\prime}(t) &=& G_{2}\left(\boldsymbol{i}+\boldsymbol{I_{0}},\boldsymbol{j}+\boldsymbol{J_{0}}, \boldsymbol{k}+\boldsymbol{K_{0}}\right) \\ \boldsymbol{k}^{\prime}(t) &=& G_{3}\left(\boldsymbol{i}+\boldsymbol{I_{0}},\boldsymbol{j}+\boldsymbol{J_{0}}, \boldsymbol{k}+\boldsymbol{K_{0}}\right) \end{array} $$

The usual tangent plane approximation on the nonlinear dynamics functions *G*_1_,*G*_2_ and *G*_3_ then gives the dynamics approximation

$$\begin{array}{@{}rcl@{}} \left[\begin{array}{c} \boldsymbol{i}^{\prime}\\ \boldsymbol{j}^{\prime}\\ \boldsymbol{k}^{\prime} \end{array}\right] \approx \left[\begin{array}{ccc} G_{1\boldsymbol{i}}^{\boldsymbol{V_{0}}} & G_{1\boldsymbol{j}}^{\boldsymbol{V_{0}}} & G_{1\boldsymbol{k}}^{\boldsymbol{V_{0}}} \\ G_{2\boldsymbol{i}}^{\boldsymbol{V_{0}}} & G_{2\boldsymbol{j}}^{\boldsymbol{V_{0}}} & G_{2\boldsymbol{k}}^{\boldsymbol{V_{0}}} \\ G_{3\boldsymbol{i}}^{\boldsymbol{V_{0}}} & G_{3\boldsymbol{j}}^{\boldsymbol{V_{0}}} & G_{3\boldsymbol{k}}^{\boldsymbol{V_{0}}} \end{array}\right] \left[\begin{array}{c} \boldsymbol{i}\\ \boldsymbol{j}\\ \boldsymbol{k} \end{array}\right] \end{array} $$

The analysis of the signs of these partials is next. This is similar to what we did for the previous model. If we hold everything constant except ***i*** which we increase to ***i***+*δ* ***i***, what happens? Increasing the IFN- *γ* level should increase IFN- *γ*.

### **Assumption 5**

For the signals ***j*** and ***k***, we assume $G_{1\boldsymbol {i}}^{\boldsymbol {V_{0}}} = +, G_{2\boldsymbol {i}}^{\boldsymbol {V_{0}}} = 0$. and $G_{3\boldsymbol {i}}^{\boldsymbol {V_{0}}} = 0$ as well.

Thus, the coefficient matrix above which we call ***Φ*** so far looks like

$$\begin{array}{@{}rcl@{}} \boldsymbol{\Phi} &=& \left[\begin{array}{ccc} + & G_{1\boldsymbol{j}}^{\boldsymbol{V_{0}}} & G_{1\boldsymbol{k}}^{\boldsymbol{V_{0}}} \\ 0 & G_{2\boldsymbol{j}}^{\boldsymbol{V_{0}}} & G_{2\boldsymbol{k}}^{\boldsymbol{V_{0}}} \\ 0 & G_{3\boldsymbol{j}}^{\boldsymbol{V_{0}}} & G_{3\boldsymbol{k}}^{\boldsymbol{V_{0}}} \end{array}\right] \end{array} $$

Now hold everything constant except ***j*** and increase ***j*** to ***j***+*δ* ***j***. What happens?

### **Assumption 6**

We assume $G_{1\boldsymbol {j}}^{\boldsymbol {V_{0}}} = +$, $G_{2\boldsymbol {j}}^{\boldsymbol {V_{0}}} = +$ and $G_{3\boldsymbol {j}}^{\boldsymbol {V_{0}}} = +$.

Thus, the coefficient matrix looks like

$$\begin{array}{@{}rcl@{}} \boldsymbol{\Phi} &=& \left[\begin{array}{ccc} + & + & G_{1\boldsymbol{k}}^{\boldsymbol{V_{0}}} \\ 0 & + & G_{2\boldsymbol{k}}^{\boldsymbol{V_{0}}} \\ 0 & + & G_{3\boldsymbol{k}}^{\boldsymbol{V_{0}}} \end{array}\right] \end{array} $$

Now hold everything constant except ***k*** and increase ***k*** to ***k***+*δ* ***k***. What happens?

### **Assumption 7**

We assume an increase in ***k*** will not effect IFN- *γ* levels, ***i***, and hence $G_{1\boldsymbol {k}}^{\boldsymbol {V_{0}}} = 0$. An increase in ***k*** must imply $G_{3\boldsymbol {k}}^{\boldsymbol {V_{0}}} = +$. We then assume the signals ***j*** and ***k*** interact so that $G_{2\boldsymbol {k}}^{\boldsymbol {V_{0}}} = -$.

Thus, the coefficient matrix now looks like

$$\begin{array}{@{}rcl@{}} \boldsymbol{\Phi} &=& \left[\begin{array}{ccc} + & + & 0 \\ 0 & + & -\\ 0 & + & + \end{array}\right] \end{array} $$

Or letting $G_{2\boldsymbol {j}}^{\boldsymbol {V_{0}}} = a, G_{3\boldsymbol {j}}^{\boldsymbol {V_{0}}} = b, G_{2\boldsymbol {k}}^{\boldsymbol {V_{0}}} = -c, G_{3\boldsymbol {k}}^{\boldsymbol {V_{0}}} = d$, we have the coefficient matrix now looks like

$$\begin{array}{@{}rcl@{}} \boldsymbol{\Phi} &=& \left[\begin{array}{ccc} + & + & 0 \\ 0 & a & -c\\ 0 & b & d \end{array}\right] \end{array} $$

### Oscillations in J and K

The eigenvalues of this linearized system are found by setting det(*λ* ***I***−***Φ***)=0. Thus, the coefficient matrix above which we call *Φ* so far looks like

$$\begin{array}{@{}rcl@{}} \det(\lambda \boldsymbol{I} - \boldsymbol{\Phi}) &=&\det \: \left[\begin{array}{ccc} \lambda - G_{1\boldsymbol{i}}^{\boldsymbol{V_{0}}}& -G_{1\boldsymbol{j}}^{\boldsymbol{V_{0}}} & -G_{1\boldsymbol{k}}^{\boldsymbol{V_{0}}} \\ 0 & \lambda - G_{2\boldsymbol{j}}^{\boldsymbol{V_{0}}} & -G_{2\boldsymbol{k}}^{\boldsymbol{V_{0}}} \\ 0 & -G_{3\boldsymbol{j}}^{\boldsymbol{V_{0}}} & \lambda - G_{3\boldsymbol{k}}^{\boldsymbol{V_{0}}} \end{array}\right] \end{array} $$

This gives

$$\begin{array}{@{}rcl@{}} 0 &=&\left(\lambda - G_{1\boldsymbol{i}}^{\boldsymbol{V_{0}}} \right) \: \det \left[\begin{array}{cc} \lambda - G_{2\boldsymbol{j}}^{\boldsymbol{V_{0}}} & -G_{2\boldsymbol{k}}^{\boldsymbol{V_{0}}} \\ -G_{3\boldsymbol{j}}^{\boldsymbol{V_{0}}} & \lambda - G_{3\boldsymbol{k}}^{\boldsymbol{V_{0}}} \end{array}\right] \end{array} $$

and so

$${} {{\begin{aligned} \det\left(\lambda \boldsymbol{I} - \boldsymbol{\Phi}\right) = \left(\lambda - G_{1\boldsymbol{i}}^{\boldsymbol{V_{0}}} \right) \: \left(\left(\lambda - G_{2\boldsymbol{j}}^{\boldsymbol{V_{0}}}\right) \left(\lambda - G_{3\boldsymbol{k}}^{\boldsymbol{V_{0}}}\right) - G_{2\boldsymbol{j}}^{\boldsymbol{V_{0}}} G_{3\boldsymbol{k}}^{\boldsymbol{V_{0}}} \right) \end{aligned}}} $$

The eigenvalues of the two by two submatrix are the most interesting. We can get complex roots if

$$\begin{array}{@{}rcl@{}} \left[\begin{array}{cc} G_{2\boldsymbol{j}}^{\boldsymbol{V_{0}}} & G_{2\boldsymbol{k}}^{\boldsymbol{V_{0}}}\\ G_{3\boldsymbol{j}}^{\boldsymbol{V_{0}}} & G_{3\boldsymbol{k}}^{\boldsymbol{V_{0}}} \end{array}\right] = \left[\begin{array}{cc} \alpha & - \beta\\ \beta & \alpha \end{array}\right] \end{array} $$

or $G_{2\boldsymbol {j}}^{\boldsymbol {V_{0}}} = G_{3\boldsymbol {k}}^{\boldsymbol {V_{0}}}$ and $G_{3\boldsymbol {j}}^{\boldsymbol {V_{0}}} = -G_{2\boldsymbol {k}}^{\boldsymbol {V_{0}}}$. The eigenvalues are then $\lambda _{1} = G_{1\boldsymbol {i}}^{o}$ and the complex conjugate pair $\alpha \pm \beta \sqrt {-1}$ where $\alpha = G_{2\boldsymbol {j}}^{\boldsymbol {V_{0}}} = G_{3\boldsymbol {k}}^{\boldsymbol {V_{0}}}$ and $\beta = G_{3\boldsymbol {j}}^{\boldsymbol {V_{0}}} = -G_{2\boldsymbol {k}}^{\boldsymbol {V_{0}}}$. The eigenvectors here are

$$\begin{aligned} &\boldsymbol{V} + \sqrt{-1} \boldsymbol{W} = \left[\begin{array}{c} 1\\ 0 \end{array}\right] + \sqrt{-1} \left[\begin{array}{c} 0 \\ 1 \end{array}\right],\\ & \boldsymbol{V} - \sqrt{-1} \boldsymbol{W} = \left[\begin{array}{c} 1\\ 0 \end{array}\right] - \sqrt{-1} \left[\begin{array}{c} 0 \\ 1 \end{array}\right] \end{aligned} $$

We can solve for ***j*** and ***k*** to find

$$\begin{array}{@{}rcl@{}} \left[\begin{array}{c} \boldsymbol{j}(t)\\ \boldsymbol{k}(t) \end{array}\right] &=& e^{\alpha t} (a \: \left(\boldsymbol{V} \: \cos(\beta t)-\boldsymbol{W} \: \sin(\beta t) \right) \: \\&& + \: b \: \left(\boldsymbol{V} \sin(\beta t)+\boldsymbol{W} \cos(\beta t)\right))\\ &=& e^{\alpha t} \: (\left(a \boldsymbol{V}+b \boldsymbol{W} \right) \: \cos(\beta t) \:\\&& + \: \left(-a \boldsymbol{W}+b \boldsymbol{V} \right) \sin(\beta t)) \end{array} $$

We then have

$${} {{\begin{aligned} \left[\begin{array}{c} \boldsymbol{j}(t)\\ \boldsymbol{k}(t) \end{array}\right] = e^{\alpha t} \left[\begin{array}{cccc} \left(a V_{1} + b W_{1} \right) \cos(\beta t) + \left(-a W_{1}+ b V_{1}\right) \sin(\beta t)\\ \left(a V_{2} + b W_{2} \right) \cos(\beta t) + \left(-a W_{2}+ b V_{2}\right) \sin(\beta t) \end{array}\right] \end{aligned}}} $$

Hence,

$$\begin{array}{@{}rcl@{}} \left[\begin{array}{c} \boldsymbol{j}(t)\\ \boldsymbol{k}(t) \end{array}\right] = e^{\alpha t} \left[\begin{array}{cc} \left(a \cos(\beta t) + b \sin(\beta t)\right.\\ \left(b \cos(\beta t) -a \sin(\beta t)\right. \end{array}\right] \end{array} $$

Letting $R = \sqrt {a^{2}+b^{2}}$, we find

$$\begin{array}{@{}rcl@{}} \left[\begin{array}{c} j(t)\\ k(t) \end{array}\right] = R e^{\alpha t} \left[\begin{array}{cc} \cos(\beta t - \delta)\\ -\sin(\beta t - \delta) \end{array}\right] \end{array} $$

where *δ* is defined as tan−1(*b*/*a*). The full solution is then

$$\begin{array}{@{}rcl@{}} \left[\begin{array}{c} \boldsymbol{i}(t) \\ \boldsymbol{j}(t)\\ \boldsymbol{k}(t) \end{array}\right] = \left[\begin{array}{c} A e^{-G_{1\boldsymbol{i}}^{o} t}\\ R e^{\alpha t} \cos(\beta t - \delta)\\ -R e^{-\alpha t} \sin(\beta t - \delta) \end{array}\right] \end{array} $$

where *A*, *R*, $G_{1\boldsymbol {i}}^{o}, \beta $ and *δ* determine a given model.

Here, we have ***J***_***0***_=*q*_1_ ***V***_***0***_ and ***K***_***0***_=(1−*p*_0_)***V***_***0***_. Hence, we roughly know at the time of the initial disturbance (infective agent or environmental toxin etc.)

$$\begin{array}{@{}rcl@{}} q_{1} \boldsymbol{V_{0}} &=& \left. \left(R e^{\alpha t} \cos(\beta t - \delta) \right. \right|_{t=0} = R \cos(\delta) \\ (1-p_{0}) \boldsymbol{V_{0}} &=& -\left. \left(R e^{-\alpha t} \sin(\beta t - \delta) \right) \right|_{t=0} = R \sin(\delta) \end{array} $$

Taking a ratio, we find

$$\begin{array}{@{}rcl@{}} \tan(\delta) &=& \frac{\boldsymbol{K_{0}}}{\boldsymbol{J_{0}}} = \frac{(1-p_{0}) \boldsymbol{V_{0}}}{q_{1} \boldsymbol{V_{0}}} = \frac{(1-p_{0})}{q_{1}}. \end{array} $$

Hence, $\delta = \tan ^{-1}\left (\frac {(1-p_{0})}{q_{1}} \right)$ and

$$\begin{array}{@{}rcl@{}} R = \boldsymbol{J_{0}} \sec(\delta) = \sqrt{\boldsymbol{K_{0}}^{2} + \boldsymbol{J_{0}}^{2}} = \boldsymbol{V_{0}} \: \sqrt{q_{1}^{2} + (1-p_{0})^{2}}. \end{array} $$

Finally, recall we have $\alpha = G_{2\boldsymbol {j}}^{\boldsymbol {V_{0}}}$ and $\beta = G_{3\boldsymbol {j}}^{\boldsymbol {V_{0}}}$; thus, the oscillatory solutions for ***j*** and ***k*** are

$$\begin{array}{@{}rcl@{}} \left[\begin{array}{c} \boldsymbol{j}(t)\\ \boldsymbol{k}(t) \end{array}\right] &=& \boldsymbol{V_{0}} \sqrt{q_{1}^{2} + (1-p_{0})^{2}} \: e^{G_{2\boldsymbol{j}}^{\boldsymbol{V_{0}}} t}\\&& \left[\begin{array}{c} \cos \left(G_{3\boldsymbol{j}}^{\boldsymbol{V_{0}}} t-\tan^{-1}\left(\frac{(1-p_{0})}{q_{1}}\right) \right)\\ -\sin \left(G_{3\boldsymbol{j}}^{\boldsymbol{V_{0}}} t-\tan^{-1}\left(\frac{(1-p_{0})}{q_{1}}\right) \right) \end{array}\right] \end{array} $$

We do not think the phase shift *δ* should be a constant; i.e. independent of ***V***_***0***_. and therefore, we assume that the critical parameters here are proportional to ***V***_***0***_. Our rough calculation showed us $R = \boldsymbol {V_{0}} \sqrt {q_{1}^{2} + (1-p_{0})^{2}}$, and thus, *R* should be proportional to ***V***_***0***_ in general. Therefore, we assume

$$\begin{array}{@{}rcl@{}} R^{\boldsymbol{V_{0}}} &\propto& \boldsymbol{V_{0}} \! \Longrightarrow R^{\boldsymbol{V_{0}}} = r_{1} \boldsymbol{V_{0}}, \quad G_{2\boldsymbol{j}}^{\boldsymbol{V_{0}}} \propto \boldsymbol{V_{0}} \Longrightarrow G_{2\boldsymbol{j}}^{\boldsymbol{V_{0}}} = r_{2} \boldsymbol{V_{0}}\\ G_{3\boldsymbol{j}}^{\boldsymbol{V_{0}}} &\propto& \boldsymbol{V_{0}} \Longrightarrow G_{3\boldsymbol{j}}^{\boldsymbol{V_{0}}} = r_{3} \boldsymbol{V_{0}}, \quad \delta^{\boldsymbol{V_{0}}} \propto \boldsymbol{V_{0}} \Longrightarrow \delta^{\boldsymbol{V_{0}}} = r_{4} \boldsymbol{V_{0}} \end{array} $$

for a new parameters *r*_1_,*r*_2_,*r*_3_ and *r*_4_. This leads to our estimate of the dependencies

$$\begin{array}{@{}rcl@{}} \left[\begin{array}{c} j(t)\\ k(t) \end{array}\right] = r_{1} \boldsymbol{V_{0}} \: e^{r_{2} \boldsymbol{V_{0}} t} \left[\begin{array}{cc} \cos \left(r_{3} \boldsymbol{V_{0}} t - r_{4} \boldsymbol{V_{0}} \right) \\ -\sin \left(r_{3} \boldsymbol{V_{0}} t - r_{4} \boldsymbol{V_{0}} \right) \end{array}\right] \end{array} $$

## A health model

Roughly speaking, if the total number of cells is ***T***, the number of healthy cells can be approximated by

$$\begin{array}{@{}rcl@{}} \boldsymbol{H} &=& \boldsymbol{T}-(C_{0} + c(t))-(M_{0}+m(t))-(N_{0}+n(t)) \end{array} $$

We know

$$\begin{array}{@{}rcl@{}} \boldsymbol{c}^{\prime} &=& H_{1\boldsymbol{i}}^{o} \: \boldsymbol{i} + H_{1\boldsymbol{j}}^{o} \: \boldsymbol{j} + H_{1\boldsymbol{k}}^{o} \: \boldsymbol{k}\\ \boldsymbol{M}^{\prime} &=& H_{2\boldsymbol{j}}^{o} \: \boldsymbol{j} + H_{2\boldsymbol{k}}^{o} \: \boldsymbol{k}\\ \boldsymbol{n}^{\prime} &=& H_{3\boldsymbol{j}}^{o} \: \boldsymbol{j} + H_{3\boldsymbol{k}}^{o} \: \boldsymbol{k} \end{array} $$

and so we are looking at deviations from the base values ***I***_***0***_=0,***J***_***0***_=*q*_1_ ***V***_***0***_ and ***K***_***0***_=(1−*p*_0_)***V***_***0***_. It follows we have

$$\begin{array}{@{}rcl@{}} \boldsymbol{C}(t) &=& \boldsymbol{C_{0}} + H_{1\boldsymbol{i}}^{o} \left(\int_{0}^{t} \boldsymbol{i}(s) ds \right) + H_{1\boldsymbol{j}}^{o} \left(q_{1} \boldsymbol{V_{0}} + \int_{0}^{t} \boldsymbol{j}(s) ds \right)\\ && + H_{1\boldsymbol{k}}^{o} \: \left((1-p_{0}) \boldsymbol{V_{0}} + \int_{0}^{t} \boldsymbol{k}(s) ds \right)\\ \boldsymbol{M}(t) &=& \boldsymbol{M_{0}} + H_{2\boldsymbol{j}}^{o} \: \left(q_{1} \boldsymbol{V_{0}} + \int_{0}^{t} \boldsymbol{j}(s) ds \right)\\ &&+ H_{2\boldsymbol{k}}^{o} \: \left((1-p_{0}) \boldsymbol{V_{0}} + \int_{0}^{t} \boldsymbol{k}(s) ds \right)\\ \boldsymbol{N}(t) &=& \boldsymbol{N_{0}} + H_{3\boldsymbol{j}}^{o} \: \left(q_{1} \boldsymbol{V_{0}} + \int_{0}^{t} \boldsymbol{j}(s) ds \right)\\&& + H_{3\boldsymbol{k}}^{o} \: \left((1-p_{0}) \boldsymbol{V_{0}} + \int_{0}^{t} \boldsymbol{k}(s) ds \right) \end{array} $$

As discussed earlier, we have initially, ***C***_***0***_=0,***M***_***0***_=*p*_2_ *p*_0_ ***V***_***0***_ and ***N***_***0***_=*p*_2_ *p*_0_ ***V***_***0***_. So we have

$$\begin{array}{@{}rcl@{}} \boldsymbol{C}(t) &=& H_{1\boldsymbol{i}}^{o} \: \left (\int_{0}^{t} \boldsymbol{i}(s) ds \right) + H_{1\boldsymbol{j}}^{o} \: \left (q_{1} \boldsymbol{V_{0}} + \int_{0}^{t} \boldsymbol{j}(s) ds \right)\\ && + H_{1\boldsymbol{k}}^{o} \: \left ((1-p_{0}) \boldsymbol{V_{0}} + \int_{0}^{t} \boldsymbol{k}(s) ds \right)\\ \boldsymbol{M}(t) &=& p_{2} \: p_{0} \: \boldsymbol{V_{0}} + H_{2\boldsymbol{j}}^{o} \: \left(q_{1} \boldsymbol{V_{0}} + \int_{0}^{t} \boldsymbol{j}(s) ds \right)\\&& + H_{2\boldsymbol{k}}^{o} \: \left((1-p_{0}) \boldsymbol{V_{0}} + \int_{0}^{t} \boldsymbol{k}(s) ds \right)\\ \boldsymbol{N}(t) &=& p_{2} \: p_{0} \: \boldsymbol{V_{0}} + H_{3\boldsymbol{j}}^{o} \: \left(q_{1} \boldsymbol{V_{0}} + \int_{0}^{t} \boldsymbol{j}(s) ds \right) \\&&+ H_{3\boldsymbol{k}}^{o} \: \left((1-p_{0}) \boldsymbol{V_{0}} + \int_{0}^{t} \boldsymbol{j}(s) ds \right) \end{array} $$

Thus, we have

$$\begin{array}{@{}rcl@{}} \boldsymbol{H}(t) &=& \boldsymbol{T} - \left(p_{1} \: p_{0} \: \boldsymbol{V_{0}} + p_{2} \: p_{0} \: \boldsymbol{V_{0}}\right) -\left(H_{1\boldsymbol{j}}^{o} + H_{2\boldsymbol{j}}^{o} + H_{3\boldsymbol{j}}^{o} \right) \: q_{1} \boldsymbol{V_{0}}\\ && -\left(H_{1\boldsymbol{k}}^{o} + H_{2\boldsymbol{k}}^{o} + H_{3\boldsymbol{k}}^{o} \right) \: (1-p_{0}) \boldsymbol{V_{0}}- H_{1\boldsymbol{i}}^{o} \: \int_{0}^{t} \boldsymbol{i}(s) ds \\ &&-\left(H_{1\boldsymbol{j}}^{o} + H_{2\boldsymbol{j}}^{o} + H_{3\boldsymbol{j}}^{o} \right) \: \int_{0}^{t} \boldsymbol{j}(s) ds\\&& - \left(H_{1\boldsymbol{k}}^{o} + H_{2\boldsymbol{k}}^{o} + H_{3\boldsymbol{k}}^{o} \right) \: \left(\int_{0}^{t} \boldsymbol{k}(s) ds \right) \end{array} $$

Now collect all the terms involving ***V***_***0***_ and set that coefficient to *Λ* for convenience. Making this replacement, we have

$$\begin{array}{@{}rcl@{}} \Lambda &=& (p_{1} + p_{2}) p_{0} + \left(H_{1\boldsymbol{j}}^{o} + H_{2\boldsymbol{j}}^{o} + H_{3\boldsymbol{j}}^{o} \right) \\&& q_{1} + \left(H_{1\boldsymbol{k}}^{o} + H_{2\boldsymbol{k}}^{o} + H_{3\boldsymbol{k}}^{o} \right) \: (1-p_{0}) \end{array} $$

This leads to the simplification

$$\begin{array}{@{}rcl@{}} \boldsymbol{H}(t) &=& \boldsymbol{T} - \Lambda \: \boldsymbol{V_{0}} - H_{1\boldsymbol{i}}^{o} \: \int_{0}^{t} \boldsymbol{i}(s) ds\\&& - \left(H_{1\boldsymbol{j}}^{o} + H_{2\boldsymbol{j}}^{o} + H_{3\boldsymbol{j}}^{o} \right) \: \int_{0}^{t} \boldsymbol{j}(s) ds\\ &&- \left(H_{1\boldsymbol{k}}^{o} + H_{2\boldsymbol{k}}^{o} + H_{3\boldsymbol{k}}^{o} \right) \: \left(\int_{0}^{t} \boldsymbol{k}(s) ds \right) \end{array} $$

Now we have to compute these integrated transient values. We label them as ***IT*** for the transient ***i*** integration; ***JT*** for the transient ***J*** integration; and ***KT*** for the transient ***K*** integration. We then have

$$\begin{array}{@{}rcl@{}} \boldsymbol{IT}(t)&=&\int_{0}^{t}\boldsymbol{i}(s) ds=\int_{0}^{t} \: A e^{-G_{1\boldsymbol{i}}^{o} s} \: ds\\ \boldsymbol{JT}(t) &=& \int_{0}^{t} \boldsymbol{j}(s) ds=\int_{0}^{t} \: R e^{\alpha t} \cos(\beta s-\delta) \: ds\\ \boldsymbol{KT}(t) &=& \int_{0}^{t} \boldsymbol{k}(s) ds = - \int_{0}^{t} \: R e^{-\alpha t} \sin(\beta s-\delta) \end{array} $$

### Integration details

The ***i*** integration is easy.

$$\begin{array}{@{}rcl@{}} \int_{0}^{t} \boldsymbol{i}(s) ds &=& \int_{0}^{t} \: A e^{-G_{1\boldsymbol{i}}^{\boldsymbol{V_{0}}} s} \: ds =\frac{A}{G_{1\boldsymbol{i}}^{\boldsymbol{V_{0}}}} \left(1 - e^{-G_{1\boldsymbol{i}}^{\boldsymbol{V_{0}}} t} \right) \end{array} $$

The ***JT*** is a standard integration by parts.

To evaluate this term, we use integration by parts. We find

$$\begin{array}{@{}rcl@{}} \boldsymbol{JT}(t) &=& \int_{0}^{t} \boldsymbol{j}(s) ds = \int_{0}^{t} \: R e^{\alpha t} \cos(\beta s - \delta) \: ds\\ &=& \frac{R}{(\alpha^{2} + \beta^{2})} \: e^{\alpha t} \left(\beta \sin(\beta t - \delta) + \alpha \cos(\beta t -\delta) \right)\\ & & + \frac{R}{\alpha^{2}+\beta^{2}} \left(\beta \sin(\delta) - \alpha \cos(\delta) \right) \end{array} $$

We can rewrite this is a much better form using our assumptions. First, rewrite as

$$\begin{array}{@{}rcl@{}} \boldsymbol{JT}(t) &=& \frac{R}{\sqrt{\alpha^{2} + \beta^{2}}} \: e^{\alpha t} \left(\frac{\beta}{\sqrt{\alpha^{2} + \beta^{2}}} \sin(\beta t - \delta)\right.\\ &&+\left. \frac{\alpha}{\sqrt{\alpha^{2} + \beta^{2}}} \cos(\beta t -\delta) \right)\\ && + \frac{R}{\sqrt{\alpha^{2}+\beta^{2}}} \left(\frac{\beta}{\sqrt{\alpha^{2} + \beta^{2}}} \sin(\delta)\right.\\ && -\left. \frac{\alpha}{\sqrt{\alpha^{2} + \beta^{2}}} \cos(\delta) \right) \end{array} $$

Now we know *α*,*β*,*δ* and *R* are really dependent of ***V***_***0***_. For convenience of exposition, let’s drop the superscript ***V***_***0***_ in our calculations below

$$\begin{array}{@{}rcl@{}} \frac{R}{\sqrt{\alpha^{2} + \beta^{2}}} &=& \frac{r_{1} \boldsymbol{V_{0}}}{\sqrt{r_{2}^{2} + r_{3}^{2}} \boldsymbol{V_{0}}} = \frac{r_{1}}{\sqrt{r_{2}^{2}+r_{3}^{2}}}, \: \frac{\alpha}{\sqrt{\alpha^{2} + \beta^{2}}}\\ &=& \frac{r_{2} \boldsymbol{V_{0}}}{\sqrt{r_{2}^{2} + r_{3}^{2}} \boldsymbol{V_{0}}} = \frac{r_{2}}{\sqrt{r_{2}^{2}+r_{3}^{2}}}\\ \frac{\beta}{\sqrt{\alpha^{2} + \beta^{2}}} &=& \frac{r_{3} \boldsymbol{V_{0}}}{\sqrt{r_{2}^{2} + r_{3}^{2}} \boldsymbol{V_{0}}} = \frac{r_{3}}{\sqrt{r_{2}^{2}+r_{3}^{2}}}, \: \delta = r_{4} \boldsymbol{V_{0}}. \end{array} $$

Finally, let’s define two new parameters, *θ*_1_ and *θ*_2_ as $\theta _{1} = \frac {r_{1}}{\sqrt {r_{2}^{2}+r_{3}^{2}}}$ and $\theta _{2} \: = \: \tan ^{-1}\left (\frac {r_{3}}{r_{2}} \right)$. Using the above, we can rewrite ***JT***(*t*) as

$$\begin{array}{@{}rcl@{}} \boldsymbol{JT}(t) &=& \theta_{1} \: e^{r_{2} \boldsymbol{V_{0}} \: t} \left(\frac{r_{3}}{\sqrt{r_{2}^{2}+r_{3}^{2}}} \sin(r_{3} \boldsymbol{V_{0}} t - r_{4} \boldsymbol{V_{0}}) \right.\\ && +\left. \frac{r_{2}}{\sqrt{r_{2}^{2}+r_{3}^{2}}} \cos(r_{3} \boldsymbol{V_{0}} t - r_{4} \boldsymbol{V_{0}}) \right)\\ && + \theta_{1} \left(\!\! \frac{r_{3}}{\sqrt{r_{2}^{2}+r_{3}^{2}}} \sin(r_{4} \boldsymbol{V_{0}}) - \frac{r_{2}}{\sqrt{r_{2}^{2}+r_{3}^{2}}} \cos(r_{4} \boldsymbol{V_{0}})\!\! \right) \end{array} $$

Using a standard reference triangle for the phase angle *θ*_2_, we see $\cos (\theta _{2}) = \frac {r_{2}}{\sqrt {r_{2}^{2}+r_{3}^{2}}}$ and $\sin (\theta _{2}) = \frac {r_{3}}{\sqrt {r_{2}^{2}+r_{3}^{2}}}$. We can then rewrite ***JT***(*t*) again as

$$\begin{array}{@{}rcl@{}} \boldsymbol{JT}(t) &=& \theta_{1} \: e^{r_{2} \boldsymbol{V_{0}} \: t} \left(\sin(\theta_{2}) \sin(r_{3} \boldsymbol{V_{0}} t - r_{4} \boldsymbol{V_{0}}) \right.\\ && + \left. \cos(\theta_{2}) \cos\left(r_{3} \boldsymbol{V_{0}} t - r_{4} \boldsymbol{V_{0}}\right) \right)\\ &&+ \theta_{1} \left(\sin(\theta_{2}) \sin(r_{4} \boldsymbol{V_{0}})-\cos(\theta_{2}) \cos(r_{4} \boldsymbol{V_{0}}) \right)\\ \end{array} $$

and using standard trigonometric identities, we then have

$$\begin{array}{@{}rcl@{}} \boldsymbol{JT}(t) &=& \theta_{1} \: e^{r_{2} \boldsymbol{V_{0}} \: t} \cos\left(r_{3} \boldsymbol{V_{0}} t - r_{4} \boldsymbol{V_{0}} - \theta_{2}\right)\\&& -\: \theta_{1} \cos\left(r_{4} \boldsymbol{V_{0}}+\theta_{2}\right) \end{array} $$

Next, another standard integration by parts shows

$$\begin{array}{@{}rcl@{}} \boldsymbol{KT}(t) &=& \int_{0}^{t} \boldsymbol{k}(s) ds= -\int_{0}^{t} \: R e^{-\alpha t} \sin(\beta s - \delta)\\ & & -\frac{R}{(\alpha^{2} + \beta^{2})} e^{\alpha t} \left (-\beta \cos(\beta t- \delta) + \alpha \sin(\beta t -\delta) \right)\\ && - \frac{R}{\alpha^{2}+\beta^{2}} \left(\beta \cos(\delta) + \alpha \sin(\delta) \right) \end{array} $$

We note the same comment on the dependence of *R*, *α*,*β* and *δ* on ***V***_***0***_ holds still. Now using these values and the terms *Q*_1_ and *Q*_2_, we we can rewrite ***KT***(*t*) as follows:

$$\begin{array}{@{}rcl@{}} \boldsymbol{KT}(t) &=& -\frac{R}{(\alpha^{2} + \beta^{2})} e^{\alpha t} \left(-\beta \cos(\beta t - \delta) \!+ \alpha \sin(\beta t -\delta)\! \right)\\ &&- \frac{R}{\alpha^{2}+\beta^{2}} \left(\beta \cos(\delta) + \alpha \sin(\delta) \right) \end{array} $$

Now using the simplifications we obtained for *α* and *β* in terms of *r*_2_ and *r*_3_, we can rewrite this complicated expression as

$$\begin{array}{@{}rcl@{}} \boldsymbol{KT}(t) = &-&\theta_{1} e^{r_{2} \boldsymbol{V_{0}} t} \left(-\frac{r_{3}}{\sqrt{r_{2}^{2}+r_{3}^{2}}} \cos(r_{3} \boldsymbol{V_{0}} t - r_{4} \boldsymbol{V_{0}}) \right.\\ &+& \left. \frac{r_{2}}{\sqrt{r_{2}^{2}+r_{3}^{2}}} \sin(r_{3} \boldsymbol{V_{0}} t - r_{4} \boldsymbol{V_{0}}) \right)\\ &-& \theta_{1} \left(\frac{r_{3}}{\sqrt{r_{2}^{2}+r_{3}^{2}}} \cos(r_{4} \boldsymbol{V_{0}}) + \frac{r_{2}}{\sqrt{r_{2}^{2}+r_{3}^{2}}} \sin(r_{4} \boldsymbol{V_{0}}) \right) \end{array} $$

Next, using the phase shift *θ*_2_, we have

$$\begin{array}{@{}rcl@{}} \boldsymbol{KT}(t) = &-&\theta_{1} \: e^{r_{2} \boldsymbol{V_{0}} t} \left(- \sin(\theta_{2}) \cos(r_{3} \boldsymbol{V_{0}} t - r_{4} \boldsymbol{V_{0}})\right. \\ &+& \left. \cos(\theta_{2}) \sin(r_{3} \boldsymbol{V_{0}} t - r_{4} \boldsymbol{V_{0}}) \right)\\ &-& \theta_{1} \: \left(\sin(\theta_{2}) \cos(r_{4} \boldsymbol{V_{0}}) + \cos(\theta_{2}) \sin(r_{4} \boldsymbol{V_{0}}) \right) \end{array} $$

This then leads to our final form

$$\begin{array}{@{}rcl@{}} \boldsymbol{KT}(t) &=& -\:\theta_{1} \: e^{r_{2} \boldsymbol{V_{0}} t} \: \sin(r_{3} \boldsymbol{V_{0}} t - r_{4} \boldsymbol{V_{0}} - \theta_{2})\\ &&-\: \theta_{1} \: \sin(r_{4} \boldsymbol{V_{0}}+ \theta_{2}) \end{array} $$

## Building the health model

Recall the health model is

$$\begin{array}{@{}rcl@{}} \boldsymbol{H}(t) &=& \boldsymbol{T} - \Lambda \: \boldsymbol{V_{0}} - H_{1\boldsymbol{i}}^{o} \: \int_{0}^{t} \boldsymbol{i}(s) ds\\&& -\left(H_{1\boldsymbol{J}}^{o} + H_{2\boldsymbol{J}}^{o} + H_{3\boldsymbol{J}}^{o} \right) \: \boldsymbol{JT}(t)\\ &&-\left(H_{1\boldsymbol{K}}^{o} + H_{2\boldsymbol{K}}^{o} + H_{3\boldsymbol{K}}^{o} \right) \: \boldsymbol{KT}(t) \end{array} $$

Let $c_{j} = H_{1\boldsymbol {J}}^{o} + H_{2\boldsymbol {J}}^{o} + H_{3\boldsymbol {J}}^{o}$ and $c_{k} = H_{1\boldsymbol {K}}^{o} + H_{2\boldsymbol {K}}^{o} + H_{3\boldsymbol {K}}^{o}$. Then we have

$$\begin{array}{@{}rcl@{}} \boldsymbol{H}(t) &=& \boldsymbol{T} - \Lambda \: \boldsymbol{V_{0}} - H_{1\boldsymbol{i}}^{o} \: \int_{0}^{t} \boldsymbol{i}(s) ds- c_{j} \: \boldsymbol{JT}(t) - c_{k} \: \boldsymbol{KT}(t) \end{array} $$

Now plug what we have found for our integrations. We have

$$\begin{array}{@{}rcl@{}} \boldsymbol{H}(t) &=& \boldsymbol{T} - \Lambda \boldsymbol{V_{0}} - H_{1\boldsymbol{i}}^{o} \frac{A}{G_{1\boldsymbol{i}}^{o}} \left(1 - e^{-G_{1\boldsymbol{i}}^{o} t} \right)\\ && -\: c_{j} \: \left\{\theta_{1} \: e^{r_{2} \boldsymbol{V_{0}} \: t} \cos(r_{3} \boldsymbol{V_{0}} t - r_{4} \boldsymbol{V_{0}} - \theta_{2})\right.\\&& -\left. \theta_{1} \cos(\theta_{2}) \cos\left(r_{4} \boldsymbol{V_{0}}+\theta_{2}\right) \vphantom{-\theta_{1} \: e^{r_{2} \boldsymbol{V_{0}} t} \: \sin(r_{3} \boldsymbol{V_{0}} t - r_{4} \boldsymbol{V_{0}} - \theta_{2})}\right\} \\ & & -\: c_{k} \: \left\{-\theta_{1} \: e^{r_{2} \boldsymbol{V_{0}} t} \: \sin(r_{3} \boldsymbol{V_{0}} t - r_{4} \boldsymbol{V_{0}} - \theta_{2})\right.\\&& -\left. \theta_{1} \: \sin\left(r_{4} \boldsymbol{V_{0}}+ \theta_{2}\right) \vphantom{-\theta_{1} \: e^{r_{2} \boldsymbol{V_{0}} t} \: \sin(r_{3} \boldsymbol{V_{0}} t - r_{4} \boldsymbol{V_{0}} - \theta_{2})}\right\} \end{array} $$

Then we can rewrite as

$$\begin{array}{@{}rcl@{}} \boldsymbol{H}(t) &=& \boldsymbol{T} - \Lambda \boldsymbol{V_{0}} - H_{1\boldsymbol{i}}^{o} \frac{A}{G_{1\boldsymbol{i}}^{o}} \left(1 - e^{-G_{1\boldsymbol{i}}^{o} t} \right)\\ && -\: \theta_{1} \: c_{j} \: \left\{ e^{r_{2} \boldsymbol{V_{0}} \: t} \cos\left(r_{3} \boldsymbol{V_{0}} t - r_{4} \boldsymbol{V_{0}} - \theta_{2}\right)\right. \\ &&-\left. \cos(r_{4} \boldsymbol{V_{0}}+\theta_{2})\vphantom{\left\{ e^{r_{2} \boldsymbol{V_{0}} \: t} \cos\left(r_{3} \boldsymbol{V_{0}} t - r_{4} \boldsymbol{V_{0}} - \theta_{2}\right)\right.}\right\} \\ & & +\: \theta_{1} \: c_{k} \: \left\{e^{r_{2} \boldsymbol{V_{0}} t} \: \sin\left(r_{3} \boldsymbol{V_{0}} t - r_{4} \boldsymbol{V_{0}} - \theta_{2}\right)\right.\\ &&+\left. \sin(r_{4} \boldsymbol{V_{0}}+ \theta_{2})\vphantom{\left\{ e^{r_{2} \boldsymbol{V_{0}} \: t} \cos\left(r_{3} \boldsymbol{V_{0}} t - r_{4} \boldsymbol{V_{0}} - \theta_{2}\right)\right.} \right\} \end{array} $$

Now put the $e^{r_{2} \boldsymbol {V_{0}} t}\phantom {\dot {i}\!}$ together. We find

$$\begin{array}{@{}rcl@{}} \boldsymbol{H}(t) &=& \boldsymbol{T} - \Lambda \boldsymbol{V_{0}} - H_{1\boldsymbol{i}}^{o} \frac{A}{G_{1\boldsymbol{i}}^{o}} \left(1 - e^{-G_{1\boldsymbol{i}}^{o} t} \right)\\ & & +\: \theta_{1} \: \left(c_{j} \: \cos\left(r_{4} \boldsymbol{V_{0}}+\theta_{2}\right) \: + \: c_{k} \: \sin\left(r_{4} \boldsymbol{V_{0}} - \theta_{2}\right) \right) \\ & & -\: \theta_{1} \: e^{r_{2} \boldsymbol{V_{0}} t} \: \left(c_{j} \: \cos\left(r_{3} \boldsymbol{V_{0}} t - r_{4} \boldsymbol{V_{0}} - \theta_{2}\right)\right. \\ &&-\left. c_{k} \: \sin\left(r_{3} \boldsymbol{V_{0}} t - r_{4} \boldsymbol{V_{0}} - \theta_{2}\right) \right) \end{array} $$

Let’s simplify some more using another phase shift. Define the phase angle $\theta _{3} = \tan ^{-1}\left (\frac {c_{j}}{c_{k}} \right)$; then, we can rewrite the health like this.

$$\begin{array}{@{}rcl@{}} \boldsymbol{H}(t) &=& \boldsymbol{T} - \Lambda \boldsymbol{V_{0}} - H_{1\boldsymbol{i}}^{o} \frac{A}{G_{1\boldsymbol{i}}^{o}} \left(1 - e^{-G_{1\boldsymbol{i}}^{o} t} \right)\\ & & -\: \theta_{1} \: e^{r_{2} \boldsymbol{V_{0}} t} \sqrt{c_{j}^{2} + c_{k}^{2}} \: \left(\frac{c_{j}}{\sqrt{c_{j}^{2} + c_{k}^{2}}} \: \cos\left(r_{3} \boldsymbol{V_{0}} t - r_{4} \boldsymbol{V_{0}} - \theta_{2}\right) \right.\\ & &\left. -\: {c_{k}}{\sqrt{c_{j}^{2} + c_{k}^{2}}} \: \sin\left(r_{3} \boldsymbol{V_{0}} t - r_{4} \boldsymbol{V_{0}} - \theta_{2}\right) \vphantom{\left(\frac{c_{j}}{\sqrt{c_{j}^{2} + c_{k}^{2}}} \: \cos\left(r_{3} \boldsymbol{V_{0}} t - r_{4} \boldsymbol{V_{0}} - \theta_{2}\right.\right)}\right) \\ & & \, +\: \theta_{1} \sqrt{c_{j}^{2} + c_{k}^{2}} \: \left(\frac{c_{j}}{\sqrt{c_{j}^{2} + c_{k}^{2}}} \: \cos\left(r_{4} \boldsymbol{V_{0}}+\theta_{2}\right)\right. \\ &&+ \left. \frac{c_{k}}{\sqrt{c_{j}^{2} + c_{k}^{2}}} \: \sin(r_{4} \boldsymbol{V_{0}} - \theta_{2}) \right) \end{array} $$

This can be recast as

$$\begin{array}{@{}rcl@{}} \boldsymbol{H}(t) &=& \boldsymbol{T} - \Lambda \boldsymbol{V_{0}} - H_{1\boldsymbol{i}}^{o} \frac{A}{G_{1\boldsymbol{i}}^{o}} \left(1 - e^{-G_{1\boldsymbol{i}}^{o} t} \right)\\ & & -\: \theta_{1} \: e^{r_{2} \boldsymbol{V_{0}} t} \sqrt{c_{j}^{2} + c_{k}^{2}} \: \left(\cos(\theta_{3}) \: \cos\left(r_{3} \boldsymbol{V_{0}} t - r_{4} \boldsymbol{V_{0}} - \theta_{2}\right)\right.\\ & &\left. - \: \sin(\theta_{3}) \: \sin\left(r_{3} \boldsymbol{V_{0}} t - r_{4} \boldsymbol{V_{0}} - \theta_{2}\right) \right) \\ & & +\: \theta_{1} \sqrt{c_{j}^{2} + c_{k}^{2}} \: \left(\cos(\theta_{3}) \: \cos\left(r_{4} \boldsymbol{V_{0}}+\theta_{2}\right)\right. \\ &&+ \left. \sin(\theta_{3}) \: \sin\left(r_{4} \boldsymbol{V_{0}} - \theta_{2}\right) \right)\\ &=& \boldsymbol{T} - \Lambda \boldsymbol{V_{0}} - H_{1\boldsymbol{i}}^{o} \frac{A}{G_{1\boldsymbol{i}}^{o}} \left(1 - e^{-G_{1\boldsymbol{i}}^{o} t} \right) \\&&-\: \theta_{1} \: e^{r_{2} \boldsymbol{V_{0}} t} \sqrt{c_{j}^{2} + c_{k}^{2}} \: \cos(r_{3} \boldsymbol{V_{0}} t\\ &&-\: r_{4} \boldsymbol{V_{0}} - \theta_{2} + \theta_{3}) + \theta_{1} \sqrt{c_{j}^{2} + c_{k}^{2}} \: \cos\left(r_{4} \boldsymbol{V_{0}} - \theta_{2} -\theta_{3}\right) \end{array} $$

Next, we can combine the ratio $H_{1\boldsymbol {i}}^{o} \frac {A}{G_{1\boldsymbol {i}}^{\boldsymbol {V_{0}}}}$ into the new parameter $\zeta _{1}^{\boldsymbol {V_{0}}}$ and rewrite $G_{1\boldsymbol {i}}^{\boldsymbol {V_{0}}}$ as $\zeta _{2}^{\boldsymbol {V_{0}}}$ to give

$$\begin{array}{@{}rcl@{}} \boldsymbol{H}(t) &=& \boldsymbol{T} - \Lambda \boldsymbol{V_{0}} - \zeta_{1}^{\boldsymbol{V_{0}}} \left(1 - e^{- \zeta_{2}^{\boldsymbol{V_{0}}} t} \right)\\&& -\: \theta_{1} \: e^{r_{2} \boldsymbol{V_{0}} t} \sqrt{c_{j}^{2} + c_{k}^{2}} \: \cos(r_{3} \boldsymbol{V_{0}} t - r_{4} \boldsymbol{V_{0}} - \theta_{2} + \theta_{3})\\ & & +\: \theta_{1} \sqrt{c_{j}^{2} + c_{k}^{2}} \: \cos(r_{4} \boldsymbol{V_{0}} - \theta_{2} -\theta_{3}) \end{array} $$

Finally, let $s_{1} = \theta _{1} \: \sqrt {c_{j}^{2} + c_{k}^{2}}$. Then, we have the last form of the health estimate:

1 $$\begin{array}{@{}rcl@{}} \boldsymbol{H}(t) &=& \boldsymbol{T} - \Lambda \boldsymbol{V_{0}} - \zeta_{1}^{\boldsymbol{V_{0}}} \left(1 - e^{- \zeta_{2}^{\boldsymbol{V_{0}}} t} \right) \\ &&-\: s_{1} e^{r_{2} \boldsymbol{V_{0}} t} \: \cos(r_{3} \boldsymbol{V_{0}} t - r_{4} \boldsymbol{V_{0}} - \theta_{2} + \theta_{3}) \\&&+\: s_{1} \cos\left(r_{4} \boldsymbol{V_{0}} - \theta_{2} -\theta_{3}\right)  \end{array} $$

We could also assume the terms $\zeta _{1}^{\boldsymbol {V_{0}}}$ and $\zeta _{2}^{\boldsymbol {V_{0}}}$ are proportional to ***V***_***0***_. We would model this by implying $\zeta _{1}^{\boldsymbol {V_{0}}} = r_{5} \boldsymbol {V_{0}}$ and $\zeta _{2}^{\boldsymbol {V_{0}}} = r_{6} \boldsymbol {V_{0}}$. We then find

2 $$\begin{array}{@{}rcl@{}} \boldsymbol{H}(t) &=& \boldsymbol{T} - \Lambda \boldsymbol{V_{0}} - r_{5} \: \boldsymbol{V_{0}} \left(1 - e^{- r_{6} \boldsymbol{V_{0}} t} \right) \\ && -\: s_{1} e^{r_{2} \boldsymbol{V_{0}} t} \: \cos\left(r_{3} \boldsymbol{V_{0}} t - r_{4} \boldsymbol{V_{0}} - \theta_{2} + \theta_{3}\right)\\ &&+\: s_{1} \cos\left(r_{4} \boldsymbol{V_{0}} - \theta_{2} -\theta_{3}\right)  \end{array} $$

These parameters depend in complex ways on the initial trigger dose ***V***_***0***_ and it is very difficult to tease out the details.

## Collateral damage

We can also work out the functional dependence on collateral damage on initial trigger dose over time. Recall the collateral damage population is given by

$$\begin{array}{@{}rcl@{}} \boldsymbol{C}(t) &=& \boldsymbol{C_{0}}+ H_{1\boldsymbol{i}}^{o} \: \left(\int_{0}^{t} \boldsymbol{i}(s) ds \right) + H_{1\boldsymbol{J}}^{o} \: \left(\! q_{1} \boldsymbol{V_{0}} + \int_{0}^{t} \boldsymbol{j}(s) ds \!\right)\\ && +\: H_{1\boldsymbol{K}}^{o} \: \left((1-p_{0}) \boldsymbol{V_{0}} + \int_{0}^{t} \boldsymbol{k}(s) ds \right)\\ &=& \boldsymbol{C_{0}}+ H_{1\boldsymbol{i}}^{o} \: \boldsymbol{IT}(t)+ H_{1\boldsymbol{j}}^{o}\: \left(q_{1} \boldsymbol{V_{0}} + \boldsymbol{JT}(t) \right)\\ &&+\: H_{1\boldsymbol{k}}^{o} \: \left((1-p_{0}) \boldsymbol{V_{0}} + \boldsymbol{KT}(t) \right) \end{array} $$

We can then substitute for ***IT***(*t*), ***JT***(*t*) and ***KT***(*t*) and obtain

$$\begin{array}{@{}rcl@{}} \boldsymbol{C}(t) &=& \boldsymbol{C_{0}} + r_{5} \boldsymbol{V_{0}} \left(1 - e^{- r_{6}\boldsymbol{V_{0}} t} \right)\\ && + H_{1\boldsymbol{j}}^{o}\: \left(q_{1} \boldsymbol{V_{0}} + \theta_{1} \: e^{r_{2} \boldsymbol{V_{0}} \: t} \cos\left(r_{3} \boldsymbol{V_{0}} t - r_{4} \boldsymbol{V_{0}} - \theta_{2}\right)\right.\\ &&-\left. \theta_{1} \cos(r_{4} \boldsymbol{V_{0}}+\theta_{2})\vphantom{\left(q_{1} \boldsymbol{V_{0}} + \theta_{1} \: e^{r_{2} \boldsymbol{V_{0}} \: t} \cos\left(r_{3} \boldsymbol{V_{0}} t - r_{4} \boldsymbol{V_{0}} - \theta_{2}\right)\right.} \right)\\ && + H_{1\boldsymbol{k}}^{o}\: \left((1-p_{0}) \boldsymbol{V_{0}} -\theta_{1} \: e^{r_{2} \boldsymbol{V_{0}} t} \: \sin\left(r_{3} \boldsymbol{V_{0}} t - r_{4} \boldsymbol{V_{0}} - \theta_{2}\! \right)\right.\\&& -\left. \theta_{1} \: \sin\left(r_{4} \boldsymbol{V_{0}}+ \theta_{2}\right)\vphantom{\left(q_{1} \boldsymbol{V_{0}} + \theta_{1} \: e^{r_{2} \boldsymbol{V_{0}} \: t} \cos\left(r_{3} \boldsymbol{V_{0}} t - r_{4} \boldsymbol{V_{0}} - \theta_{2}\right)\right.}\!\! \right) \end{array} $$

Now, collect terms as we did in our earlier simplifications. We rewrite as

$$\begin{array}{@{}rcl@{}} \boldsymbol{C}(t) &=& \boldsymbol{C_{0}}+r_{5} \boldsymbol{V_{0}} \left(\!1-e^{- r_{6}\boldsymbol{V_{0}} t}\! \right) + \left(H_{1\boldsymbol{k}}^{o} \: (1-p_{0}) + H_{1\boldsymbol{j}}^{o} \: q_{1}\right) \! \boldsymbol{V_{0}}\\ &&+ \:\theta_{1} \: e^{r_{2} \boldsymbol{V_{0}} t} \left(H_{1\boldsymbol{j}}^{o}\:\cos\left(r_{3} \boldsymbol{V_{0}} t - r_{4} \boldsymbol{V_{0}} - \theta_{2}\right)\right. \\&&- \left. H_{1\boldsymbol{k}}^{o}\: \sin\left(r_{3} \boldsymbol{V_{0}} t - r_{4} \boldsymbol{V_{0}} - \theta_{2}\right)\vphantom{\left(H_{1\boldsymbol{j}}^{o}\:\cos\left(r_{3} \boldsymbol{V_{0}} t - r_{4} \boldsymbol{V_{0}} - \theta_{2}\right)\right.}\! \right)\\ &&-\: \theta_{1} \left(H_{1\boldsymbol{j}}^{o}\: \cos\left(r_{4} \boldsymbol{V_{0}}+\theta_{2}\right) +H_{1\boldsymbol{k}}^{o}\: \sin\left(r_{4} \boldsymbol{V_{0}}+ \theta_{2}\right) \right) \end{array} $$

We can also introduce an additional phase shift, *phi*, as follows. It will be different from the phase shift

$$\begin{array}{@{}rcl@{}} \theta_{3} &=& \tan^{-1}\left(\frac{c_{j}}{c_{k}}\right) = \tan^{-1} \left(\frac{H_{1\boldsymbol{j}}^{o}+H_{2\boldsymbol{j}}^{o}+H_{3\boldsymbol{j}}^{o}} {H_{1\boldsymbol{k}}^{o}+H_{2\boldsymbol{k}}^{o}+H_{3\boldsymbol{k}}^{o}} \right) \end{array} $$

as here we only use the *H*_1_ partials: $\phi =\tan ^{-1} \left (\frac {H_{1\boldsymbol {k}}^{o}}{H_{1\boldsymbol {j}}^{o}} \right)$. We rewrite as

$$\begin{array}{@{}rcl@{}} \boldsymbol{C}(t) &=& \boldsymbol{C_{0}} + r_{5} \boldsymbol{V_{0}} \left(1-e^{- r_{6}\boldsymbol{V_{0}} t} \right) + \left(H_{1\boldsymbol{k}}^{o} \: (1-p_{0}) + H_{1\boldsymbol{j}}^{o} \: q_{1}\right) \: \boldsymbol{V_{0}}\\ &&+\: \theta_{1} \: \sqrt{\left(H_{1\boldsymbol{j}}^{o}\right)^{2} +\left(H_{1\boldsymbol{k}}^{o}\right)^{2}}\:e^{r_{2} \boldsymbol{V_{0}} t} \left(\cos(\phi) \:\cos\left(r_{3} \boldsymbol{V_{0}} t - r_{4} \boldsymbol{V_{0}} - \theta_{2}\right)\right.\\ && - \left. \sin(\phi) \: \sin\left(r_{3} \boldsymbol{V_{0}} t - r_{4} \boldsymbol{V_{0}} - \theta_{2}\right) \right)\\ && -\: \theta_{1}\: \sqrt{\left(H_{1\boldsymbol{j}}^{o}\right)^{2}+\left(H_{1\boldsymbol{k}}^{o}\right)^{2}}\: (\cos(\phi) \: \cos\left(r_{4} \boldsymbol{V_{0}}+\theta_{2}\right) \\&& +\: \sin(\phi) \: \sin\left(r_{4} \boldsymbol{V_{0}}+ \theta_{2}\right)) \end{array} $$

We can then use the the usual cos laws of addition and subtraction of angles to repackage this as

$$\begin{array}{@{}rcl@{}} \boldsymbol{C}(t) &=& \boldsymbol{C_{0}}+ r_{5} \boldsymbol{V_{0}} \left(1 - e^{- r_{6}\boldsymbol{V_{0}} t} \right)+ \left(H_{1\boldsymbol{k}}^{o} \: (1-p_{0}) + H_{1\boldsymbol{j}}^{o} \: q_{1}\right) \: \boldsymbol{V_{0}}\\ &&+ \theta_{1} \: \sqrt{\left(H_{1\boldsymbol{j}}^{o}\right)^{2}\,+\,\left(H_{1\boldsymbol{k}}^{o}\right)^{2}}\:e^{r_{2} \boldsymbol{V_{0}} t} \cos\left(r_{3} \boldsymbol{V_{0}} t - r_{4} \boldsymbol{V_{0}} - \theta_{2}+\phi\right)\\ & &- \theta_{1}\: \sqrt{\left(H_{1\boldsymbol{j}}^{o}\right)^{2}+\left(H_{1\boldsymbol{k}}^{o}\right)^{2}}\: \cos\left(r_{4} \boldsymbol{V_{0}}+\theta_{2}-\phi\right) \end{array} $$

Now define $s_{2} = \theta _{1}\: \sqrt {(H_{1\boldsymbol {j}}^{o})^{2}+(H_{1\boldsymbol {k}}^{o})^{2}}$ and rewrite as

$$\begin{array}{@{}rcl@{}} \boldsymbol{C}(t) &=& \boldsymbol{C_{0}}+ r_{5} \boldsymbol{V_{0}} \left(1 - e^{- r_{6}\boldsymbol{V_{0}} t} \right) + \left(H_{1\boldsymbol{k}}^{o} \: (1-p_{0}) + H_{1\boldsymbol{j}}^{o} \: q_{1}\right) \: \boldsymbol{V_{0}}\\ &&+\: s_{2} \:e^{r_{2} \boldsymbol{V_{0}} t} \cos\left(r_{3} \boldsymbol{V_{0}} t - r_{4} \boldsymbol{V_{0}} - \theta_{2}+\phi\right)\\ && -~ s_{2} \: \cos\left(r_{4} \boldsymbol{V_{0}}+\theta_{2}-\phi\right) \end{array} $$

Since collateral damage is initially zero, we have as our final form

$$\begin{array}{@{}rcl@{}} \boldsymbol{C}(t) &=& r_{5} \boldsymbol{V_{0}} \left(1 - e^{- r_{6}\boldsymbol{V_{0}} t} \right) + \left(H_{1\boldsymbol{k}}^{o} \: (1-p_{0}) + H_{1\boldsymbol{j}}^{o} \: q_{1}\right) \boldsymbol{V_{0}}\\ &&+\: s_{2} \:e^{r_{2} \boldsymbol{V_{0}} t} \cos\left(r_{3} \boldsymbol{V_{0}} t - r_{4} \boldsymbol{V_{0}} - \theta_{2}+\phi\right)\\ &&-\: s_{2} \: \cos\left(r_{4} \boldsymbol{V_{0}}+\theta_{2}-\phi\right) \end{array} $$

Previously, we used the simplification

$$\begin{array}{@{}rcl@{}} \Lambda &=& (p_{1} + p_{2}) p_{0}z + \left(H_{1\boldsymbol{j}}^{o} + H_{2\boldsymbol{j}}^{o} + H_{3\boldsymbol{j}}^{o} \right) \: q_{1}\\ &&+\left(H_{1\boldsymbol{k}}^{o} + H_{2\boldsymbol{k}}^{o} + H_{3\boldsymbol{k}}^{o} \right) \: (1-p_{0}) \end{array} $$

This needs to be modified to

$$\begin{array}{@{}rcl@{}} \Lambda^{1} &=& H_{1\boldsymbol{j}}^{o} \: q_{1} +H_{1\boldsymbol{k}}^{o} \: (1-p_{0}). \end{array} $$

Our final collateral damage function is then

$$\begin{array}{@{}rcl@{}} \boldsymbol{C}(t) &=& \Lambda^{1} \boldsymbol{V_{0}} + r_{5} \boldsymbol{V_{0}} \left(1 - e^{- r_{6}\boldsymbol{V_{0}} t} \right)\\ &&+\; s_{2} e^{r_{2} \boldsymbol{V_{0}} t} \cos\left(r_{3} \boldsymbol{V_{0}} t - r_{4} \boldsymbol{V_{0}} - \theta_{2}+\phi\right)\\ && -\; s_{2} \cos\left(r_{4} \boldsymbol{V_{0}}+\theta_{2}-\phi\right) \end{array} $$

**Fig. 1:**
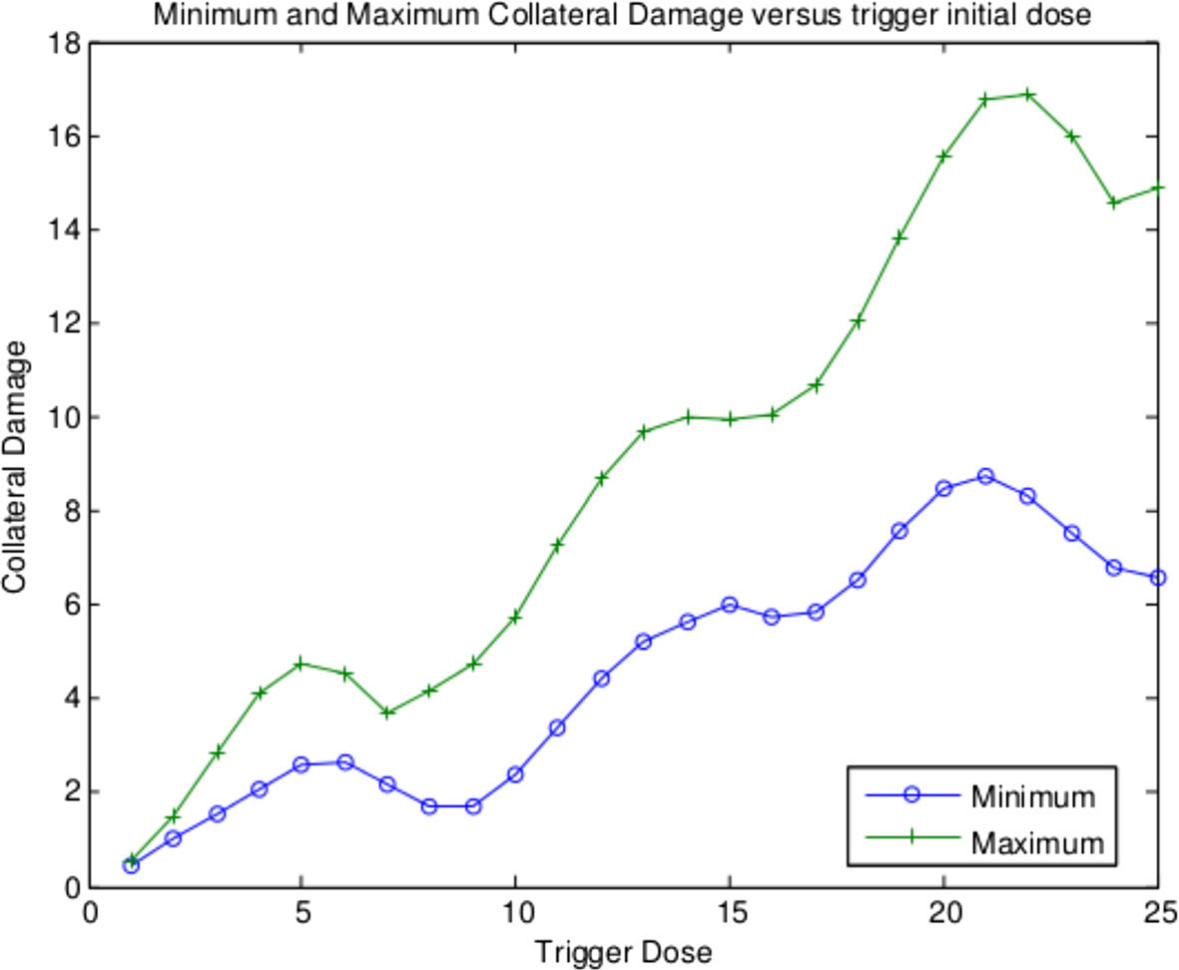
Collateral damage versus trigger dose

Of course, since *S*_2_, *Λ* ^1^ and *ϕ* are different from the corresponding values in the health function, it is a bit difficult to compare simulation results, but it is easy to see the qualitative ideas of oscillation in health and collateral. We have done similar experiments in [3] and indeed the graphs we now show were generated using the same MatLab code. The point is that the existence of the oscillation in health, collateral damage and so forth is due to the assumptions we made on the nonlinear interactions between the two populations ***M*** and ***N*** mediated by the signals ***J*** and ***K***. As long as those sorts of interactions are occurring, this kind of interaction behavior is assured; and that is *very* interesting we feel. We can easily run a quick simulation to see if our predictions of oscillations in the health and collateral damage function are verified. We use the same parameter settings and MatLab code as we used in [3]. The interested reader can look those details up as necessary. We ran the simulation with the chosen parameter values from [3] and plotted both the maximum and minimum collateral values versus the trigger dose in Fig. 1.

Note that there is variation in the collateral damage due to the nonlinear interactions between the ***J***, and ***K***. This is due to the assumptions we have made on the kinds of nonlinear interactions that occur. The remainder of this paper will necessarily discuss why we think these kind of nonlinearities are possible in a variety of auto immune situations.

As noted in [3], it is clear what is happening. The model

$$\begin{array}{@{}rcl@{}} \boldsymbol{H}(t) &=& \boldsymbol{T} - \Lambda \boldsymbol{V_{0}} - r_{5} \: \boldsymbol{V_{0}} \left(1 - e^{- r_{6} \boldsymbol{V_{0}} t} \right) \\ &&-\: s_{1} e^{r_{2} \boldsymbol{V_{0}} t} \: \cos\left(r_{3} \boldsymbol{V_{0}} t - r_{4} \boldsymbol{V_{0}} - \theta_{2} + \theta_{3}\right)\\ &&+\: s_{1} \cos\left(r_{4} \boldsymbol{V_{0}} - \theta_{2} -\theta_{3}\right) \end{array} $$

can be written in terms of **decay** and **push - pull** terms as follows:

$$\begin{array}{@{}rcl@{}} - \Lambda \boldsymbol{V_{0}} &=& \mathbf{decay }\\ - r_{5} \: \boldsymbol{V_{0}} \left(1 - e^{- r_{6} \boldsymbol{V_{0}} t} \right) &=& \mathbf{decay}\\ - s_{1} e^{r_{2} \boldsymbol{V_{0}} t} \: \cos\left(r_{3} \boldsymbol{V_{0}} t - r_{4} \boldsymbol{V_{0}} - \theta_{2} + \theta_{3}\right) \\ + s_{1} \cos\left(r_{4} \boldsymbol{V_{0}} - \theta_{2} -\theta_{3}\right) &=& \mathbf{push - pull } \end{array} $$

Thus, we have ***H***(*t*) always decreases unless the **push - pull** terms counteract that decay. Hence, what is important is the term

$$\begin{array}{@{}rcl@{}} \boldsymbol{\Delta}(t) &=& -\: s_{1} e^{r_{2} \boldsymbol{V_{0}} t} \: \cos\left(r_{3} \boldsymbol{V_{0}} t - r_{4} \boldsymbol{V_{0}} - \theta_{2} + \theta_{3}\right)\\ &&+\: s_{1} \cos\left(r_{4} \boldsymbol{V_{0}} - \theta_{2} -\theta_{3}\right) \end{array} $$

can oscillate as trigger load increases. To do this, it is important for the two terms in ***Δ***(*t*) to be out of phase. Hence, roughly speaking cos(*r*_3_ ***V***_***0***_ *t*−*r*_4_ ***V***_***0***_−*θ*_2_+*θ*_3_) must be sometimes negative when cos(*r*_4_ ***V***_***0***_−*θ*_2_−*θ*_3_) is positive. This allows for an increase in health of approximately *ξ* *s*_1_ where *ξ* is the difference between the two terms. This is possible when the two cos arguments are out of phase by about *π* radians. Note it is also important that the exponential term $\phantom {\dot {i}\!}e^{r_{2} \boldsymbol {V_{0}} t}$ allows growth. The interaction dynamics are determined by

$$\begin{array}{@{}rcl@{}} \left[\begin{array}{cc} G_{2\boldsymbol{j}}^{\boldsymbol{V_{0}}} & G_{2\boldsymbol{k}}^{\boldsymbol{V_{0}}}\\ G_{3\boldsymbol{j}}^{\boldsymbol{V_{0}}} & G_{3\boldsymbol{k}}^{\boldsymbol{V_{0}}} \end{array}\right] = \left[\begin{array}{cc} \alpha & - \beta\\ \beta & \alpha \end{array}\right] \end{array} $$

and we have argued that the appropriate algebraic signs for this coefficient matrix $\boldsymbol {\mathcal {M}}$ are

$$\begin{array}{@{}rcl@{}} \boldsymbol{\mathcal{M}} &=& \left[\begin{array}{cc} + & - \\ + & + \end{array}\right] \end{array} $$

We can have complex eigenvalues and hence oscillating behavior if the signs were

$$\begin{array}{@{}rcl@{}} \boldsymbol{\mathcal{M}} &=& \left[\begin{array}{cc} - & + \\ - & - \end{array}\right] \end{array} $$

but then the real part of the eigenvalues would be negative and we would have to model the exponential term as $e^{-r_{2} \boldsymbol {V_{0}} t}\phantom {\dot {i}\!}$. The induced oscillations would then be damped it would not be possible to see oscillatory grown in the collateral damage function.

**Fig. 2:**
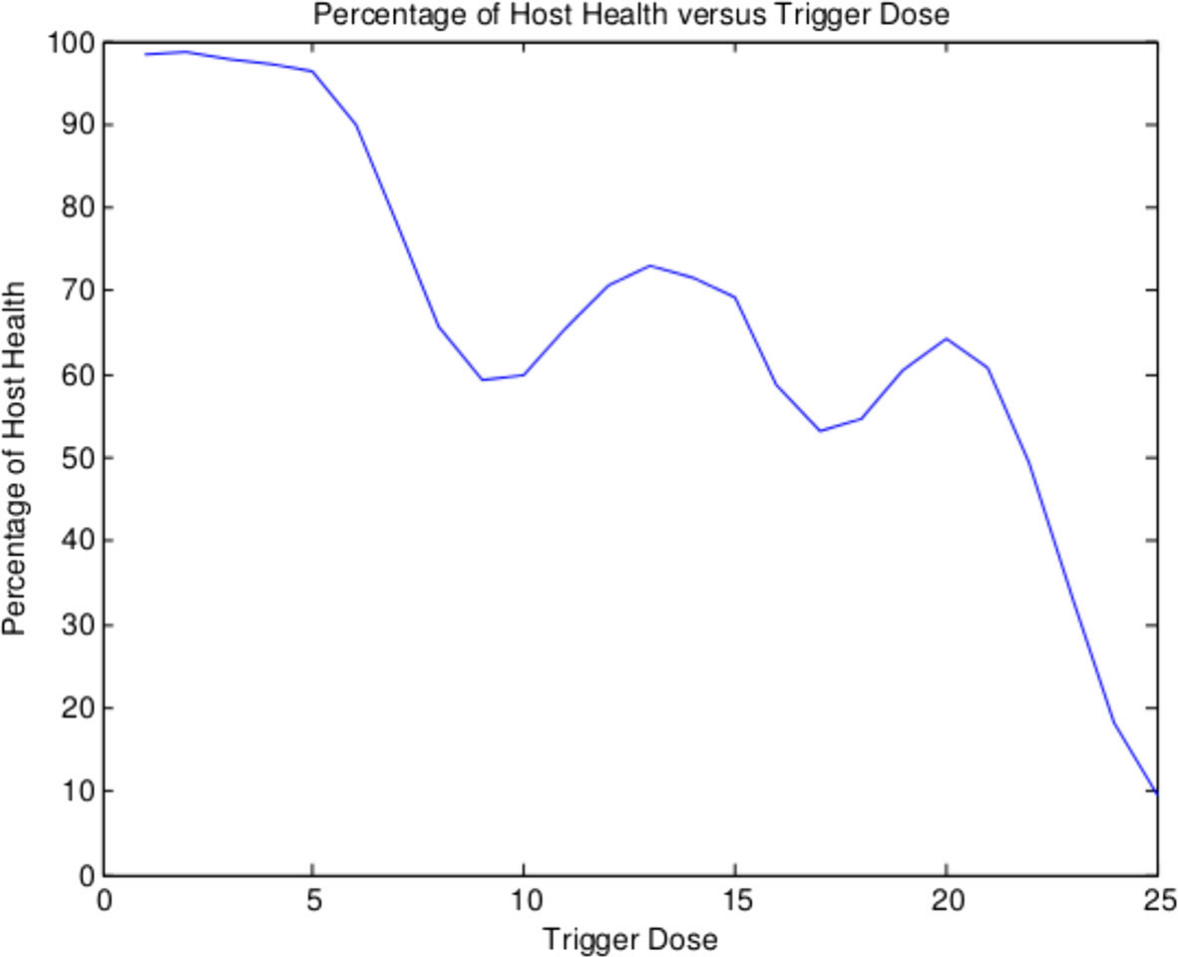
Minimal heath percentage versus trigger dose

Note we can also plot the minimum health obtained over the simulation time for each trigger dose as we did in [3]. We find the percentage minimal values are shown in Fig. 2.

**Fig. 3:**
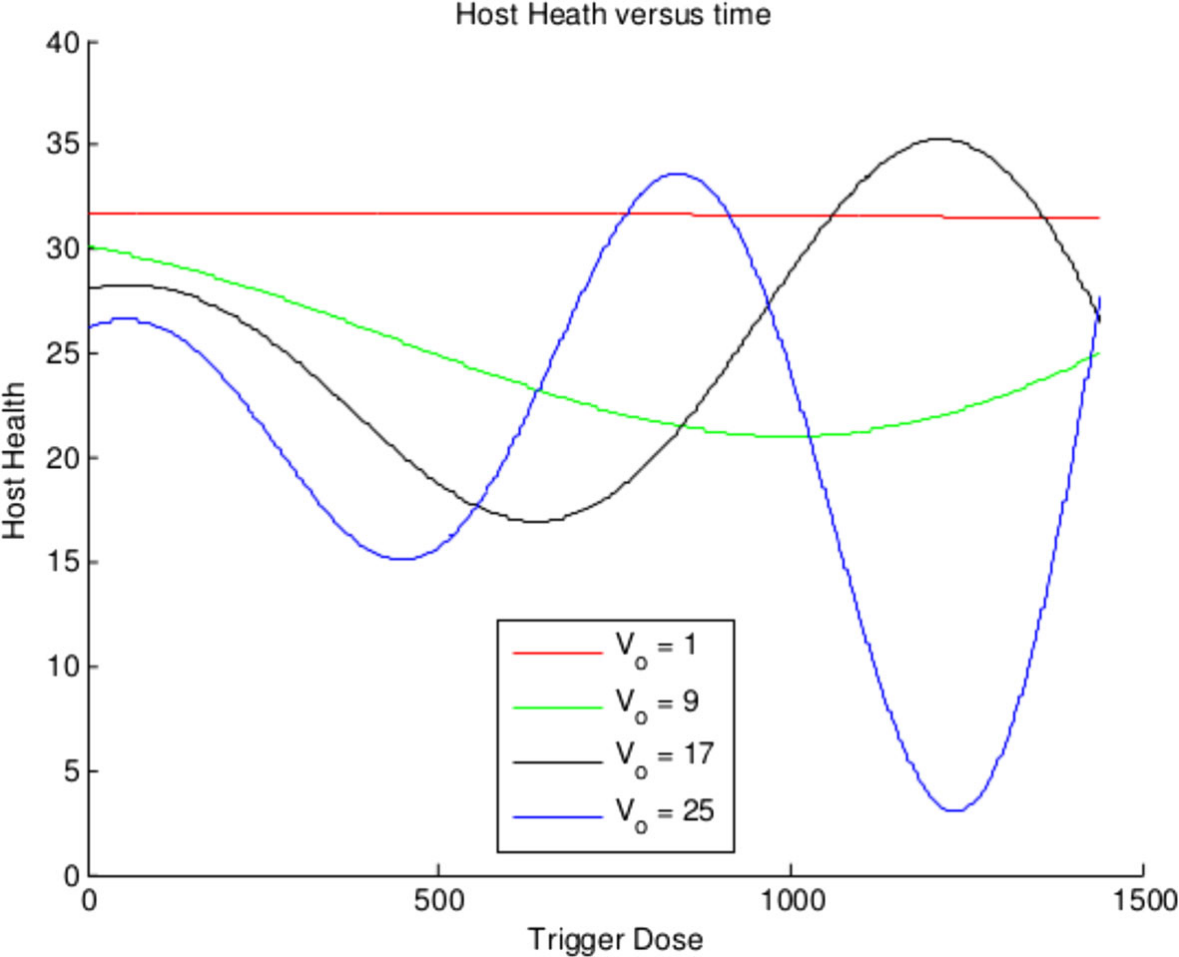
Four health versus time plots for different initial trigger doses

Let’s take a moment to reflect on what we are seeing here. If we assume a trigger has an effect on the host’s cell population that leads to two distinct cell populations ***M*** and ***N*** that interact in nonlinear ways following Assumption 1 - Assumption 7 due to the mediating influences of two signals ***J*** and ***K***, we inevitably see the host healthy cell population over time have up and down variations due to the size of initial trigger ***V***_***o***_. Consider the health plots for four levels of ***V***_***o***_ shown in Fig. 3.

Note that the minimal values of health do indeed decline for increasing initial trigger dose (although we see up and down variation if we look at all the minimal values versus initial trigger dose as we show in Fig. 1. What we want to focus on here is that the health at many initial trigger doses oscillates over the time of the simulation. For example, the health plot corresponding to ***V***_***o***_=25 has a very low minimum value. These plots are generated by abstractions of health and collateral damage and so it is not clear at all how to relate them to the health of a real host, but it does suggest that the host health can *rebound* from a low value. Hence, collateral damage can diminish for a given initial trigger which shows a kind of *relapse* effect.

Also note, the theoretical model we have built so far generates what we call collateral damage and relates it to general health with no discussion of T Cell recognition of infected cells. For us collateral damage is related to IFN- *γ* signalling which is generated by the lysis of cells in the host. In the West Nile Virus infection studied in [1], the splitting into two separate cell populations due to the WNV antigen causes explicitly changes in the avidity computation of the T Cells that recognize MHC-1 complexes on infected cells. These changes lead to an enhanced probability that T Cells will target healthy cells because *self* proteins become more visible to the adaptive immune system. In [3], we derive the heath and collateral damage model we also develop in this paper by focusing very explicitly on the splitting phenomena. The possibility of self damage is now redirected much more strongly to the splitting into two cell populations which allows for a much more general treatment of self damage. However, it is now time to think more deeply about what the signals ***J*** and ***K*** would be in specific cases of auto immune response to a trigger.

## General trigger models

We now want to think carefully about the signals ***J*** and ***K*** and the trigger ***V***. Hence, we consider the transcriptional control of a regulatory molecule which can be co-opted by an external trigger. A good example is the regulatory molecule *N* *F* *κ* *B* whose normal action is changed by the WNV antigen. It always plays a role in immune system response but the virus alters what it does in many ways. We have discussed this sort of modeling in [4] for the purpose of approximating computation in excitable neurons and in that paper, we follow the spirit of the semi-abstract approach in [5]. We are now going to re-task it for our purposes of an auto immune discussion, however, this same way of looking at signalling for the context of altering computation in a cognitive model provides another way of looking at the same idea and we think it is always useful to examine a complicated mechanism from multiple points of view.

Consider a trigger *T*_0_ which activates a cell surface receptor. Inside the cell, there are always protein kinases that can be activated in a variety of ways. Here we denote a protein kinase by the symbol *PK*. A common mechanism for such an activation is to add to *PK* another protein subunit  to form the complex . This chain of events looks like this:  where CSR denotes a *cell surface receptor*.  then acts to *phosphorylate* another protein. The cell is filled with large amounts of a transcription factor we will denote by *T*_1_ and an inhibitory protein for *T*_1_ we label as $T_{1}^{\sim }$. This symbol, $T_{1}^{\sim }$, denotes the *complement* or *anti* version of *T*_1_. In the cell, *T*_1_ and $T_{1}^{\sim }$ are generally joined together in a complex denoted by $T_{1}/T_{1}^{\sim }$. The addition of $T_{1}^{\sim }$ to *T*_1_ prevents *T*_1_ from being able to access the genome in the nucleus to transcribe its target protein. Using methods similar to those discussed in [4], we find the effects of the trigger *T*_0_ on the change in protein production $T_{1}, \delta _{T_{1}}\phantom {\dot {i}\!}$, can be modeled by



for *β*>>1. From this quick analysis, we can clearly see the potentially explosive effect changes in *T*_0_ can have on .

We can use these results for our general immune interaction discussion. We have assumed there are two different cell populations ***M*** and ***N*** which are effected differently by the trigger. Hence, let’s assume there is a pathway involving potentially many steps that leads to an alteration of the *fragility* of these two cell populations. In a West Nile Virus infection, this fragility is related to the upregulation of MHC-1 complexes but it could be another type of alteration of the overall health of the cell. Let the pathway leading to a change in fragility for ***M*** be $\boldsymbol {\mathcal {P_{M}}}$ and the one leading to the fragility alteration in ***N*** be $\boldsymbol {\mathcal {P_{N}}}$. Then associated such a change in fragility with the alteration of a protein or protein complex we call *T*_*M*_ or *T*_*N*_ depending on the cell population type. Then we have

$$\begin{array}{@{}rcl@{}} \delta_{T_{M}} &=& \mu_{M} \: \left(2 \epsilon_{M}\: + \: \epsilon_{M}^{2}\right) \quad \text{and} \quad \delta_{T_{N}} = \mu_{N} \: \left(2 \epsilon_{N}\: + \: \epsilon_{N}^{2}\right) \end{array} $$

for parameters *μ*_*M*_, *μ*_*N*_, *ε*_*M*_ and *ε*_*N*_. Hence, we can estimate a strength level for the trigger effect on each population that leads to increases in fragility.

### Computational abstractions

**Fig. 4:**
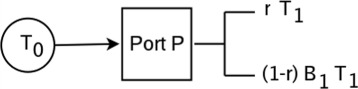
A second messenger trigger

**Fig. 5:**
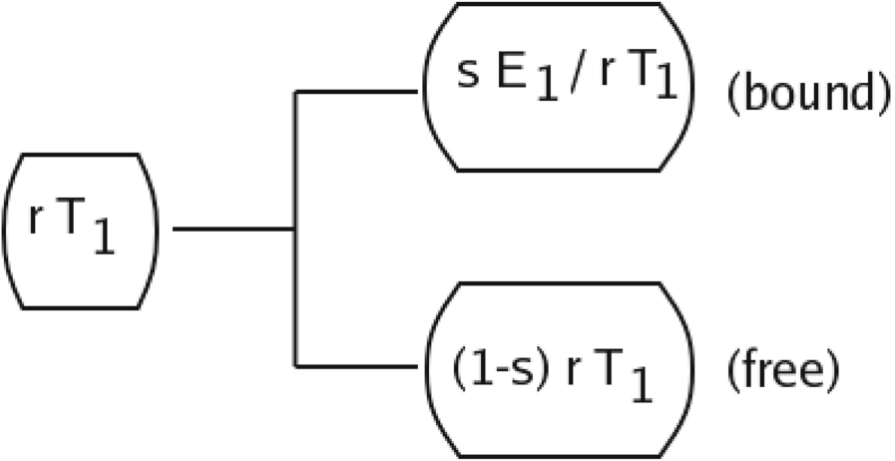
Some *T*_1_ binds to the genome

A close look extracellular triggers abstractly helps us understand how to approximate their effects. Let *T*_0_ denote a second messenger trigger which moves though a port *P* to create a new trigger *T*_1_ some of which binds to *B*_1_. A schematic of this is shown in Fig. 4. In the figure, *r* is a number between 0 and 1 which represents the fraction of the trigger *T*_1_ which is free in the cytosol. Hence, 100*r*% of *T*_1_ is free and 100(1−*r*) is bound to *B*_1_ creating a storage complex *B*_1_/*T*_1_. For our simple model, we assume *r* *T*_1_ is transported to the nuclear membrane where some of it binds to the enzyme *E*_1_. Let *s* in (0,1) denote the fraction of *r* *T*_1_ that binds to *E*_1_. We illustrate this in Fig. 5.

We denote the complex formed by the binding of *E*_1_ and *T*_1_ by *E*_1_/*T*_1_. From Fig. 5, we see that the proportion of *T*_1_ that binds to the genome (DNA) and initiates protein creation *P*(*T*_1_) is thus *s* *r* *T*_1_.

The protein created, *P*(*T*_1_), could be many things. Here, let us assume that *P*(*T*_1_) is a protein that binds to the promoter for one of the many proteins that effect MHC-1 creation, peptide binding etc. For our purposes, we will call it $\boldsymbol {\mathcal {Q}}$. Thus, our high level model is $sE_{1}/rT_{1} \: + \: \text {DNA} \rightarrow \boldsymbol {\mathcal {Q}}$. We therefore increase the concentration of MHC-I complexes on the surface of the call, thereby making the cell more visible to the adaptive immune system.

**Fig. 6:**
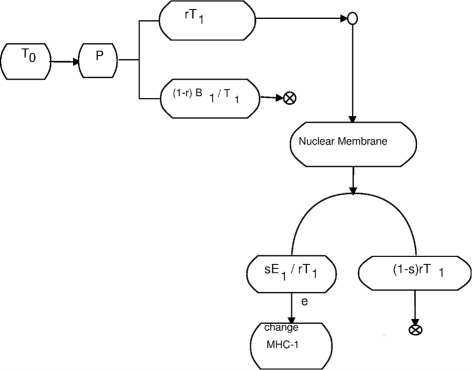
MHC-1 complex pathway

We can model increases in $\boldsymbol {\mathcal {Q}}$ as increases in T Cell binding efficiency, where *e* is a number between 0 and 1. We will not assume all of the *s* *E*_1_/*r* *T*_1_ + DNA to ***M*** reaction is completed. It follows that *e* is similar to a Michaelson - Mentin kinetics constant. Our full schematic is then given in Fig. 6.

We can model the choice process, *r* *T*_1_ or (1−*r*)*B*_1_/*T*_1_ via a simple sigmoid, $f(x) = 0.5 (1 + \tanh (\frac {x-x_{0}}{g}))$ where the transition rate at *x*_0_ is $f^{\prime }(x_{0}) = \frac {1}{2g}$. Hence, the “gain” of the transition can be adjusted by changing the value of *g*. We assume *g* is positive. This function can be interpreted as switching from of “low” state **0** to a high state **1** at speed $\frac {1}{2g}$. Now the function *h*=*r* *f* provides an output in (*r*,*∞*). If *x* is larger than the threshold *x*_0_, *h* rapidly transitions to a high state *r*. On the other hand, if *x* is below threshold, the output remains near the low state 0.

We assume the trigger *T*_0_ does not activate the port *P* unless its concentrations is past some threshold [*T*_0_]_*b*_ where [*T*_0_]_*b*_ denotes the *base* concentration. Hence, we can model the port activity by $h_{p}([T_{0}]) = \frac {r}{2} \left ({\vphantom {\frac {[T_{0}]-[T_{0}]_{b}}{g_{p}}}1 + \tanh }\right.\left.\left (\frac {[T_{0}]-[T_{0}]_{b}}{g_{p}}\right)\right)$ where the two shaping parameters *g*_*p*_ (transition rate) and [*T*_0_]_*b*_ (threshold) must be chosen. We can thus model the schematic of Fig. 4 as *h*_*p*_([*T*_0_]) [*T*_1_]_*n*_ where [*T*_1_]_*n*_ is the nominal concentration of the induced trigger *T*_1_. In a similar way, we let $h_{e}(x) = \frac {s}{2}(1 + \tanh (\frac {x-x_{0}}{g_{e}}))$ Thus, for *x* = *h*_*p*_([*T*_0_]) [*T*_1_]_*n*_, we have *h*_*e*_ is a switch from 0 to *s*. Note that 0 ≤ *x* ≤ *r*[*T*_1_]_*n*_ and so if *h*_*p*_([*T*_0_]) [*T*_1_]_*n*_ is close to *r*[*T*_1_]_*n*_,*h*_*e*_ is approximately *s*. Further, if *h*_*p*_([*T*_0_]) [*T*_1_]_*n*_ is small, we will have *h*_*e*_ is close to 0. This suggests a threshold value for *h*_*e*_ of $\frac {r[T_{1}]_{n}}{2}$. We conclude

$${} {{\begin{aligned} h_{e} \left(h_{p}([T_{0}]) \: [T_{1}]_{n}\right) = \frac{s}{2} \left(~ 1 + \tanh\left(\frac{h_{p}([T_{0}])[T_{1}]_{n} -\frac{r[T_{1}]_{n}}{2}}{g_{e}}\right) \right) \end{aligned}}} $$

which lies in [0,*s*). This is the amount of activated *T*_1_ which reaches the genome to create the target protein *P*(*T*_1_). It follows then that [*P*(*T*_1_)]=*h*_*e*_(*h*_*p*_([*T*_0_])[*T*_1_]_*n*_)[*T*_1_]_*n*_. The protein is created with efficiency *e* and so we model the conversion of [*P*(*T*_1_)] into a change in $\boldsymbol {\mathcal {Q}}$ as follows. Let

$$\begin{array}{@{}rcl@{}} h_{\boldsymbol{\mathcal{Q}}}(x) &=& \frac{e}{2} \left(~1 + \tanh\left(\frac{x-x_{0}}{ g_{\boldsymbol{\mathcal{Q}}}}\right) ~ \right) \end{array} $$

which has output in [0,*e*). Here, we want to limit how large a change we can achieve in $\boldsymbol {\mathcal {Q}}$. Hence, we assume there is an upper limit which is given by $\Delta \: \boldsymbol {\mathcal {Q}} \: = \: \delta _{\boldsymbol {\mathcal {Q}}} \: \boldsymbol {\mathcal {Q}}^{max}$. Thus, we limit the change in the the expression of $\boldsymbol {\mathcal {Q}}$ to some percentage of a baseline value. It follows that $h_{\boldsymbol {\mathcal {Q}}}(x)$ is about $\delta _{\boldsymbol {\mathcal {Q}}}$ if *x* is sufficiently large and small otherwise. This suggests that *x* should be [*P*(*T*_1_)] and since translation to *P*(*T*_1_) occurs no matter how low [*T*_1_] is, we can use a switch point value of *x*_0_=0. We conclude

3 $$\begin{array}{@{}rcl@{}}{} h_{\boldsymbol{\mathcal{Q}}}(\left[P(T_{1})\right]) = \frac{e}{2} \: \delta_{\boldsymbol{\mathcal{Q}}} \: \boldsymbol{\mathcal{Q}}^{max} \left(~1 + \tanh\left(\frac{\left[P(T_{1})\right]}{ g_{\boldsymbol{\mathcal{Q}}}}\right)\right)  \end{array} $$

Our model of the change in $\boldsymbol {\mathcal {Q}}$ expression is therefore $\Delta \: \boldsymbol {\mathcal {Q}}^{max} \: = \: h_{\boldsymbol {\mathcal {Q}}}(\left [P(T_{1})\right ])$.

We can use these results for our general immune interaction discussion as well. From earlier comments, we know that associated with a change in fragility in the two cell populations ***M*** and ***N*** is the alteration of a protein or protein complex called *P*(*T*_*M*_) or *P*(*T*_*N*_) depending on the cell population type. Then we have

$$\begin{array}{@{}rcl@{}} h_{\boldsymbol{M}}(\left[P(T_{M})\right]) &=& \frac{e_{P(T_{M})}}{2} \: \delta_{P(T_{M})} \: \boldsymbol{P(T_{M})}^{max} \\&& \left(1 + \tanh\left(\frac{\left[P(T_{M})\right]}{g_{\boldsymbol{P(T_{M})}}}\right)\right) \end{array} $$

Our model of the change in maximum *P*(*T*_*M*_) expression is therefore $\Delta \: \boldsymbol {P(T_{M})}^{max} \: = \: h_{\boldsymbol {P(T_{M})}}(\left [P(T_{M})\right ])$. We can do this for the ***N*** cells as well and obtain

$$\begin{array}{@{}rcl@{}} h_{\boldsymbol{N}}(\left[P(T_{N})\right]) &=& \frac{e_{P(T_{N})}}{2} \: \delta_{P(T_{N})} \: \boldsymbol{P(T_{N})}^{max}\\&& \left(1 + \tanh\left(\frac{\left[P(T_{N})\right]}{g_{\boldsymbol{P(T_{N})}}}\right) \right) \end{array} $$

Our model of the change in maximum *P*(*T*_*N*_) expression is therefore $\Delta \: \boldsymbol {P(T_{N})}^{max} \: = \: h_{\boldsymbol {P(T_{N})}}([P(T_{N})])$.

## Diffusion trigger models

We will now look at the trigger from a diffusion perspective. This is different as in the earlier models, we focus on how cell populations change in time and derive health and collateral functions that show their dependence on the initial viral dose. We did not consider any spatial relationships between the cellular populations. Now we will do so and the discussion will give us another way to look at the nonlinear interactions between ***M*** and ***N***. It is well known that second messenger systems often involve *C* *a* ^++^ ion movement in and out of the cell. The amount of free *C* *a* ^++^ ion in the cell is controlled by complicated mechanisms, but some is stored in buffer complexes. The release of calcium ion from these buffers plays a big role in cellular regulatory processes and which protein *P*(*T*_1_) is actually created from a trigger *T*_0_. The diffusion model is very powerful. Consider some substance *u* which satisfies

$$\begin{array}{@{}rcl@{}} \frac{\partial u }{ \partial t } &=& D \frac{\partial^{2} {u} }{ \partial {x}^{2} }\\ -D \frac{\partial u }{ \partial x } \mid_{0,L} &=& J_{0,L} \end{array} $$

where *D* is called the diffusion constant for this substance. For simplicity this is a one dimensional model where the spatial variable *x* comes from the line segment [0,*L*]. For example, instead of a three dimensional cell modeled as a sphere, the cell is modeled as a string of finite length. Hence, stuff can only enter the *cell* from either the right or the left. The condition $-D \frac {\partial u }{ \partial x } \mid _{0,L} = J_{0,L}$ states that there are conditions on the flux of *u* through the boundary at *x*=−0 or *x*=*L* that must be satisfied. The term *J*_0,*L*_ can be thought of as an injection of current into the cell. In this model, we think of the substance *u* as being in equilibrium in the cell and the current injection *J*_0,*L*_ alters that equilibrium and the diffusion model, when solved, tells us what happens to *u* due to the sudden current injection at the boundary. The critical review on the control of free calcium in cellular processing in [6] notes the concentration of *C* *a* ^++^ in the cell is controlled by the reversible binding of calcium ion to the buffer complexes, *B*_1_,*B*_2_ and so forth. There in general are quite a few different buffer complexes which all behave differently. These buffer molecules act as calcium ion sensors that, in a sense, decode the information contained in the calcium ion current injection and then pass on a decision to a target. Many of these targets can be proteins transcribed by accessing the genome. Hence, the *P*(*T*_1_) could be a buffer molecule *B*_*j*_ and so the trigger that causes the calcium current injection into the cell could influence the concentration of a buffer *B*_*j*_ and therefore influence how it itself is decoded. In this situation, the boundary condition *J*_0,*L*_ plays the role of the entry calcium current. Such a calcium ion input current through the membrane could be due to membrane depolarization causing an influx of calcium ions through the port or via ligand binding to a receptor which in turn indirectly increases free calcium ion in the cytosol. Such mechanisms involve the interplay between the way the endoplasmic reticulum handles release and uptake and also the storage buffers. This boundary current in general therefore determines the *u*(*t*,*x*) solution through the diffusion equation. The exact nature of this solution is determined by the receptor types, buffers and storage sites in the ER. Differences in the period and magnitude of the calcium current *u*(*t*,*x*) resulting from the input *J*_0,*L*_ trigger different second messenger pathways. Hence, there are many possible outcomes due to a given input current *J*_0,*L*_.

We will now modify discussions in [7, 8] and [9] that show us how to model calcium ion movement in the cell to develop a model of trigger movement in and out of the cell populations ***M*** and ***N***.

We assume the trigger enters the host and can be sequestered in some form in both ***M*** and ***N*** cell populations. We also assume the trigger has associated with some sort of diffusion process; letting *u*(*t*,*x*) be the concentration of the trigger in the host at time *t* and spatial position *x*, we posit *u*_*t*_=*D*_0_ *u*_*xx*_. Also, note we present our arguments as if the host was one dimensional; i.e., the two cell populations lies along a one dimensional axis measured by the location variable *x*. This is, of course, very simplistic, but we only want to suggest some functional dependencies here, so it will suffice for our purposes. Let’s assume these two populations use or bind the trigger with binding rate constants $k_{M}^{+}$ and $k_{N}^{+}$ and disassociation rate constants $k_{M}^{-} $ and $k_{N}^{-}$. Let the total number of cells in the host be *P*. Then the fraction of cells in the ***M*** population is $\frac {\boldsymbol {M}}{P}$ which we call *C*_*M*_ and the fraction in the ***N*** population is $\frac {\boldsymbol {M}}{P}$ which is denoted by *C*_*N*_. Hence, the number of cells that have not been exposed to the trigger is ***P***−***M***(*t*,*x*)−***N***(*t*,*x*)=***F***(*t*,*x*).

Now, let *u*(*t*,*x*) be the concentration of free trigger in the host at (*t*,*x*). Some of the trigger has been used to create cells in the populations ***M*** and ***N***, but the rest is unused. Hence, if ***C*** is a cell which has not been altered by the trigger, we have the reactions

$$\begin{array}{@{}rcl@{}} T + \: \boldsymbol{C} &\rightarrow_{k_{M}^{+}}& \boldsymbol{M}, \quad \boldsymbol{M} \rightarrow_{k_{M}^{-}} T \: + \: \boldsymbol{C}\\ T + \: \boldsymbol{C} &\rightarrow_{k_{N}^{+}}& \boldsymbol{N}, \quad \boldsymbol{N} \rightarrow_{k_{N}^{-}} T \: + \: \boldsymbol{C} \end{array} $$

where *T* is the trigger. Of course, the equations above depend on time and space, but we have not written in that dependence to avoid clutter. Note also, in this context, the backward reaction in which trigger is freed from the cellular populations ***M***(*t*,*x*) and ***N***(*t*,*x*) is typically that of lysis and so the backward rates are part of our nonlinear interaction model. We are just adding low level detail. The corresponding dynamics are

$$\begin{array}{@{}rcl@{}} \frac{d\left[T\right]}{dt} &=& - \: k_{M}^{+} \left[T\right]\left[\boldsymbol{C}\right] \: + \: k_{M}^{-} \left[\boldsymbol{M}\right],\\ \quad \frac{d\left[\boldsymbol{M}\right]}{dt} &=& \: k_{M}^{+} \left[T\right] \left[\boldsymbol{C}\right] \: - \: k_{M}^{-} \left[\boldsymbol{M}\right]\\ \frac{d\left[T\right]}{dt} &=& - \: k_{N}^{+} \left[T\right]\left[\boldsymbol{C}\right] \: + \: k_{N}^{-} \left[\boldsymbol{N}\right],\\ \quad \frac{d\left[\boldsymbol{N}\right]}{dt} &=& \: k_{N}^{+} \left[T\right] \left[\boldsymbol{N}\right] \: - \: k_{N}^{-} \left[\boldsymbol{N}\right] \end{array} $$

and we also know $\left [\boldsymbol {C}\right ] = \frac {\boldsymbol {P} - \boldsymbol {M} - \boldsymbol {N}}{\boldsymbol {P}}$. The amount of trigger being freed from the ***M*** population is $k_{M}^{-} \frac {\boldsymbol {M}}{\boldsymbol {P}} = k_{M}^{-} C_{M}$ and the amount being added to the ***M*** population is the amount of trigger not in the ***M*** state minus the amount of trigger already in the ***M*** state. This can be calculated at

$$\begin{array}{@{}rcl@{}} k_{M}^{+} \: u(t,x) \: \frac{\boldsymbol{P} - \boldsymbol{N}}{\boldsymbol{P}} \: - \: k_{M}^{+} \frac{\boldsymbol{M}}{\boldsymbol{P}} u(t,x) &=& k_{M}^{+} \left(\!\frac{\boldsymbol{P} - \boldsymbol{N}}{\boldsymbol{P}} - \frac{\boldsymbol{M}}{\boldsymbol{P}}\! \right) u(t,x) \end{array} $$

To make the manipulations easier, let $B_{M} = 1 - \frac {\boldsymbol {N}}{\boldsymbol {P}}$ and $B_{N} = 1 - \frac {\boldsymbol {M}}{\boldsymbol {P}}$. We can rewrite the equation above as

$$\begin{array}{@{}rcl@{}} k_{M}^{+} \left(\frac{\boldsymbol{P} - \boldsymbol{N}}{\boldsymbol{P}} - \frac{\boldsymbol{M}}{\boldsymbol{P}} \right) u(t,x) &=& k_{M}^{+} \left(B_{M} - C_{M} \right) u(t,x) \end{array} $$

A similar analysis gives the amount of trigger being added to the ***N*** population as

$$\begin{array}{@{}rcl@{}} k_{N}^{+} \left(\frac{\boldsymbol{P} - \boldsymbol{M}}{\boldsymbol{P}} - \frac{\boldsymbol{N}}{\boldsymbol{P}} \right) u(t,x) &=& k_{N}^{+} \left(B_{N} - C_{N} \right) u(t,x) \end{array} $$

Thus, the diffusion dynamics are

$$\begin{array}{@{}rcl@{}} \frac{\partial u}{\partial t} &=& k_{M}^{-} C_{M} - k_{M}^{+} \left(B_{M} - C_{M} \right) u(t,x)\\&& +\: k_{N}^{-} C_{N} - k_{N}^{+} \left(B_{N} - C_{N} \right) u(t,x) + D_{0} \frac{\partial^{2} u}{\partial x^{2}} \end{array} $$

where *D*_0_ is diffusion coefficient for free trigger. We now assume the spread of cells we collect into the populations ***M*** and ***N*** satisfy some sort of diffusion law. Certainly, cells are added to these populations as the trigger diffuses throughout the host’s body. Hence, this assumption is a good start. We therefore assume the diffusion dynamics for *C*_*M*_ and *C*_*N*_ are given by

$$\begin{array}{@{}rcl@{}} \frac{\partial C_{M}}{\partial t} &=& - k_{M}^{-} C_{M} \: + \: k_{M}^{+} \left(B_{M} - C_{M} \right) u(t,x) + D_{M} \frac{\partial^{2} C_{M}}{\partial x^{2}}\\ \frac{\partial C_{N}}{\partial t} &=& - k_{N}^{-} C_{N} \: + \: k_{N}^{+} \left(B_{N} - C_{N} \right) u(t,x) + D_{N} \frac{\partial^{2} C_{N}}{\partial x^{2}} \end{array} $$

Now consider the free trigger plus a correction due to the population fractions *C*_*M*_ and *C*_*N*_. We denote this by *w*(*t*,*x*) and note that *w*=*u* + *C*_*M*_+*C*_*N*_ and

$$\begin{array}{@{}rcl@{}} w_{xx} &=& u_{xx} + (C_{M})_{xx} + (C_{N})_{xx} \: \Longrightarrow u_{xx}\\ &=& w_{xx} - (C_{M})_{xx} - (C_{N})_{xx}. \end{array} $$

Thus,

$$\begin{array}{@{}rcl@{}} \frac{\partial w}{\partial t} &=& u_{t} + (C_{M})_{t} + (C_{N})_{t} = k_{M}^{-} C_{M}\\&& - k_{M}^{+} (B_{M} - C_{M}) (w - C_{M} - C_{N})\\&& + k_{N}^{-} C_{N} - k_{N}^{+} (B_{N} - C_{N}) (w - C_{M} - C_{N}) + D_{0} \: u_{xx}\\ & & -k_{M}^{-} C_{M} + k_{M}^{+} (B_{M} - C_{M}) (w - C_{M} - C_{N}) + D_{M} (C_{M})_{xx} \\ & & - k_{N}^{-} C_{N} + k_{N}^{+} (B_{N} - C_{N}) (w - C_{M} - C_{N}) + D_{N} (C_{M})_{xx} \end{array} $$

This simplifies to

$$\begin{array}{@{}rcl@{}} \frac{\partial w}{\partial t} &=& D_{0} \:u_{xx} + D_{M} (C_{M})_{xx} + D_{N} (C_{N})_{xx}\\ &=& D_{0} \: (w_{xx} - (C_{M})_{xx} - (C_{N})_{xx}) + D_{M} (C_{M})_{xx}\\ &&+\: D_{N} (C_{N})_{xx}\\ &=& D_{0} \: w_{xx} + (D_{0} - D_{M}) (C_{M})_{xx} + (D_{0} - D_{N}) (C_{N})_{xx} \end{array} $$

Thus, *w* satisfies

4 $$\begin{array}{@{}rcl@{}} w_{t} &=& D_{0} w_{xx} + (D_{M} - D_{0}) (C_{M})_{xx}\\ &&+ (D_{N} - D_{0}) (C_{N})_{xx}, -D_{0} w_{x}\left|{\!~\!}_{0,L} = J_{0,L}\right. \end{array} $$

where we have not shown the derivation of the boundary terms here as they are less germane to our interests. It seems reasonable to assume that the interaction with the cell populations, determined by $k_{M}^{-}$ and $k_{M}^{+} u$ is *fast* and reaches equilibrium quickly. Hence, we will assume that (*C*_*M*_)_*t*_=(*C*_*M*_)_*xx*_=0 and (*C*_*N*_)_*t*_=(*C*_*N*_)_*xx*_=0 giving

$$\begin{array}{@{}rcl@{}} -k_{M}^{-} C_{M} = k_{M}^{+} \left(B_{M} - C_{M} \right) u, \quad -k_{N}^{-} C_{N} = k_{N}^{+} \left(B_{N} - C_{N} \right) u \end{array} $$

Solving, we find

$$\begin{array}{@{}rcl@{}} C_{M} &=& \frac{k_{M}^{+} B_{M} u}{k_{M}^{-} + k_{M}^{+} u}\\ C_{N} &=& \frac{k_{N}^{+} B_{N} u}{k_{N}^{-} + k_{N}^{+} u} \end{array} $$

Now define $K_{M} = \frac {}{}$ and $K_{N} = \frac {}{}$. We can then rewrite our equations as

5 $$\begin{array}{@{}rcl@{}} C_{M} &=& \frac{B_{M} u}{K_{M} + u} \Longrightarrow C_{M} (K_{M} + u) = B_{M} u \end{array} $$

6 $$\begin{array}{@{}rcl@{}}  C_{N} &=& \frac{B_{N} u}{K_{N} + u} \Longrightarrow C_{N} (K_{N} + u) = B_{N} u \end{array} $$

Plugging in for *u*, we have

$$\begin{array}{@{}rcl@{}} C_{M} K_{M} &+& (C_{M} - B_{M}) (w - C_{M} - C_{N}),\\ \quad C_{N} K_{N}&+& (C_{N} - B_{N}) (w - C_{M} - C_{N}) \end{array} $$

Then, another rewrite gives

$$\begin{array}{@{}rcl@{}} B_{M} w &=& C_{M} (K_{M} + B_{M} + w) - C_{M}^{2} + B_{M} C_{N}\\ B_{N} w &=& C_{N} (K_{N} + B_{N} + w) - C_{N}^{2} + B_{N} C_{M} \end{array} $$

Note this tells us that *C*_*M*_ and *C*_*N*_ are functions of the *w* Let *C*_*M*_(*w*) and *C*_*N*_(*w*)denote this functional dependence. From the chain rule, we have $\frac {\partial C_{M} }{ \partial x } = \frac {\partial C_{M} }{ \partial w } \: \frac {\partial w }{ \partial x }$ and $\frac {\partial C_{N} }{ \partial x } = \frac {\partial C_{N} }{ \partial w } \: \frac {\partial w }{ \partial x }$. Then, the dynamics become

$$\begin{array}{@{}rcl@{}} w_{t} &=& \frac{\partial^{2} {w} }{ \partial {x}^{2} } + \frac{\partial}{\partial x} \left(\! (D_{M} - D_{0}) \frac{\partial C_{M} }{ \partial w } \frac{\partial w }{ \partial x } + (D_{N} - D_{0}) \frac{\partial C_{N} }{ \partial w } \frac{\partial w }{ \partial x } \right)\\ &=& \frac{\partial}{\partial x} \left(\left(D_{0} + (D_{M} - D_{0}) \frac{\partial C_{M} }{ \partial w } + (D_{N} - D_{0}) \frac{\partial C_{N} }{ \partial w } \right) \frac{\partial w }{ \partial x } \right) \end{array} $$

Notice that if we define a new diffusion coefficient,  for the diffusion process that governs *w* by

7 $$\begin{array}{@{}rcl@{}} \mathcal{D} &=& D_{0} + (D_{M}- D_{0}) \frac{\partial C_{M} }{ \partial w } ++ (D_{N}- D_{0}) \frac{\partial C_{N} }{ \partial w } \end{array} $$

we obtain



### Further approximations

What would it mean if *u*≪*K*_*M*_ and *u*≪*K*_*N*_? Let’s take the first case: *u*≪*K*_*M*_ implies $k_{M}^{+} u \ll k_{M}^{-}$. Now $k_{M}^{-}$ represents the rate that the ***M*** cells lose the trigger. This occurs only when the cells are destroyed. These cells are destroyed either because they have exceeded their normal lifespans or because the trigger has made them more fragile. This fragility could mean the cells have caught the attention of the immune system and are being destroyed or the fragility of the cells is such that standard apoptosis strategies are employed to remove the damaged cell. Note losing trigger from the ***M*** cells therefore corresponds to increasing trigger concentration. Since $k_{M}^{+}$ is the rate at which ***M*** cells are formed, $k_{M}^{+} u$ giving the amount of trigger lost from the formation of the ***M*** cells per ***M*** cell. We generally assume that in a trigger situation, the trigger is growing inside the ***M*** and ***N*** cell populations. If the trigger is a virus, replication only occurs after infection and once inside these cells, the virus grows. This additional trigger is then released into the host upon lysis of a ***M*** or ***N*** cell. It seems reasonable to assume the amount of released trigger is usually quite a bit bigger than the amount of trigger that initiates the formation of these cells. Thus, the inequality $k_{M}^{+} u \ll k_{M}^{-}$ seems reasonable. A similar argument shows that $k_{N}^{+} u \ll k_{N}^{-}$. Thus, it is reasonable to assume *u*≪*K*_*m*_ and *u*≪*K*_*N*_ which leads to the approximations

8 $$\begin{array}{@{}rcl@{}} C_{M} &=& \frac{B_{M}}{K_{M}} \: u, \quad C_{N} = \frac{B_{N}}{K_{N}} \: u \end{array} $$

Thus, *w* has become

$$\begin{array}{@{}rcl@{}} w &=& \left(1 + \frac{B_{M}}{K_{M}} + \frac{B_{N}}{K_{N}} \right) \: u \Longrightarrow u = \frac{w}{1 + \frac{B_{M}}{K_{M}} + \frac{B_{N}}{K_{N}}} \end{array} $$

We can then rewrite *C*_*M*_ and *C*_*N*_ as

$$\begin{array}{@{}rcl@{}} C_{M} &=& \frac{B_{M}}{K_{M}} \: \frac{w}{1 + \frac{B_{M}}{K_{M}} + \frac{B_{N}}{K_{N}}}, \quad \quad C_{N} = \frac{B_{N}}{K_{N}} \: \frac{w}{1 + \frac{B_{M}}{K_{M}} + \frac{B_{N}}{K_{N}}} \end{array} $$

or

$$\begin{array}{@{}rcl@{}} C_{M} &=& \frac{\frac{B_{M}}{K_{M}} } { 1 + \frac{B_{M}}{K_{M}} + \frac{B_{N}}{K_{N}}} \: w, \quad \quad C_{N} = \frac{\frac{B_{N}}{K_{N}}} { 1 + \frac{B_{M}}{K_{M}} + \frac{B_{N}}{K_{N}}} \: w \end{array} $$

We can find the partials with respect to *w*:

$$\begin{array}{@{}rcl@{}} (C_{M})_{w} &=& \frac{\frac{B_{M}}{K_{M}}}{1 + \frac{B_{M}}{K_{M}} + \frac{B_{N}}{K_{N}}}, \quad \quad (C_{N})_{w} = \frac{\frac{B_{N}}{K_{N}}}{ 1 + \frac{B_{M}}{K_{M}} + \frac{B_{N}}{K_{N}} } \end{array} $$

Then, letting $\gamma _{M}= \frac {B_{M}}{K_{M}}$ and $\gamma _{N} = \frac {B_{N}}{K_{N}}$, we have

$$\begin{array}{@{}rcl@{}} (C_{M})_{w} &=& \frac{\gamma_{M}}{1 + \gamma_{M} + \gamma_{N}}, \quad \quad (C_{N})_{w} = \frac{\gamma_{N}}{ 1 + \gamma_{M} + \gamma_{N}} \end{array} $$

We can then redo our calculations for *w*_*t*_.

$$\begin{array}{@{}rcl@{}} w_{t} &=& \frac{\partial}{\partial x} \left\{ \left(D_{0} + (D_{M} - D_{0}) \left(\frac{\gamma_{M}}{1 + \gamma_{M} + \gamma_{N}}\right)\right.\right.\\ &&+ \left. \left.(D_{N} - D_{0}) \left(\frac{\gamma_{N}}{ 1 + \gamma_{M} + \gamma_{N}}\right) \right) \frac{\partial w }{ \partial x } \right\} \end{array} $$

Letting *Λ* denote the term 1+*γ*_*M*_+*γ*_*N*_, then *w*=*Λ* *u* and so we have

$$\begin{array}{@{}rcl@{}} w_{t} &=& \Lambda u_{t}, \quad \quad w_{x} = \Lambda u_{x}, \quad \quad w_{xx} = \Lambda u_{xx}. \end{array} $$

We also have

$$\begin{array}{@{}rcl@{}} w_{t} &=& \frac{D_{0} + D_{M} \gamma_{M} + D_{N} \gamma_{N}}{\Lambda} \: w_{xx}\\ &=& (D_{0} + D_{M} \gamma_{M} + D_{N} \gamma_{N}) u_{xx} \end{array} $$

Thus,

$$\begin{array}{@{}rcl@{}} \Lambda u_{t} &=& (D_{0} + D_{M} \gamma_{M} + D_{N} \gamma_{N}) \: u_{xx} \Longrightarrow u_{t}\\ &=& \frac{\Lambda \: D_{0} + D_{M} \gamma_{M} + D_{N} \gamma_{N}} {\Lambda} \: u_{xx} \end{array} $$

Define the new diffusion constant $\hat {\mathcal {D}}$ by $\hat {\mathcal {D}} = \frac {D_{0} + D_{M} \gamma _{M} + D_{N} \gamma _{N}} {\Lambda }$. The free trigger dynamics are thus .

### Approximations to trigger modification

Let’s examine what might happen if a trigger event *T*_0_ initiated an increase in ***M***. This trigger event initiates a complex pathway culminating in a protein transcription (see Section “General trigger models” for the general trigger discussion). Recall, we let the pathway leading to a change in fragility for ***M*** be $\boldsymbol {\mathcal {P_{M}}}$. We associate such a change in fragility with the alteration of a subsidiary signal *T*_*M*_ and we derived

$$\begin{array}{@{}rcl@{}} \delta_{T_{M}} &=& \mu_{M} \: \left(2 \epsilon_{M}\: + \: \epsilon_{M}^{2}\right) \end{array} $$

for parameters *μ*_*M*_ and *ε*_*M*_. In Section “Computational abstractions”, a computational approach using sigmoid activation function *h*_***M***_ further found if the change in fragility in ***M*** due to the signal *T*_*M*_ is the alteration of a protein or protein complex called *P*(*T*_*M*_), then

$$\begin{array}{@{}rcl@{}} h_{\boldsymbol{M}}(\left[P(T_{M})\right]) &=& \frac{e_{P(T_{M})}}{2} \: \delta_{P(T_{M})} \: \boldsymbol{P(T_{M})}^{max}\\ && \left(1 + \tanh\left(\frac{\left[P(T_{M})\right]}{g_{\boldsymbol{P(T_{M})}}}\right) \right) \end{array} $$

where $e_{P(T_{M})}$ is a scaling factor and $\delta _{P(T_{M})}$ is the change in *P*(*T*_*M*_) expression. Our model of the change in maximum *P*(*T*_*M*_) expression is therefore $\Delta \: \boldsymbol {P(T_{M})}^{max} \: = \: h_{\boldsymbol {P(T_{M})}}([P(T_{M})])$.

Let’s assume *C*_*M*_ is increased to *C*_*M*_+*ε*. It is reasonable to assume that both $k_{M}^{+}$ and $k_{M}^{-}$ are independent of the amount of *C*_*M*_ that is present. The same comment holds for $k_{N}^{+}$ and $k_{N}^{-}$. We have $B_{M} = 1 - \frac {N}{P}$ stays the same, but $B_{N} = 1 - \frac {M}{P} = 1 - C_{M} \rightarrow 1 - C_{M} - \epsilon = B_{N} - \epsilon $. Thus,

$$\begin{array}{@{}rcl@{}} \Lambda &=& 1 + \frac{B_{M}}{K_{M}} + \frac{B_{N}}{K_{M}} \rightarrow 1 + \frac{B_{M}}{K_{M}} + \frac{B_{N}}{K_{N}} - \frac{\epsilon}{K_{N}}. \end{array} $$

Thus, the new value is $\hat {\Lambda } = \Lambda - \frac {\epsilon }{K_{N}}$. This implies

$$\begin{array}{@{}rcl@{}} \hat{\mathcal{D}} &=& \frac{D_{0} + D_{M} \frac{B_{M}}{K_{M}} + D_{N} \frac{B_{N}}{K_{N}} }{\Lambda} \rightarrow \frac{D_{0} + D_{M} \frac{B_{M}}{K_{M}} + D_{N} \frac{B_{N}}{K_{N}} - D_{N} \frac{\epsilon}{K_{N}}}{\Lambda- \frac{\epsilon}{K_{N}}} \end{array} $$

$\tilde {\mathcal {D}} = \frac {\Lambda \: \hat {\mathcal {D}} - \epsilon \frac {D_{N}}{K_{N}}}{\Lambda - \epsilon \frac {1}{K_{N}}}$. Now let *ξ*_*M*_ denote $\frac {1}{K_{N}}$ and use that in the equation above. We find $\tilde {\mathcal {D}} = \frac {\Lambda \: \hat {\mathcal {D}} - \epsilon \xi _{M} D_{N} }{\Lambda - \epsilon \xi _{M} }$. The change in the diffusion constant is then

$$\begin{array}{@{}rcl@{}} \Delta \hat{\mathcal{D}} &=&\hat{\mathcal{D}} - \frac{\Lambda \: \hat{\mathcal{D}} - \epsilon \xi_{M} D_{N} }{\Lambda - \epsilon \xi_{M} } = \frac{\epsilon D_{N}}{\Lambda - \epsilon \xi_{M}} \: \left(1 - \frac{\hat{\mathcal{D}}}{D_{N}} \right) \end{array} $$

To first order, we know $\frac {\epsilon }{\Lambda - \epsilon \xi _{M}} \approx \frac {\epsilon }{\Lambda }$ and so $\Delta \hat {\mathcal {D}} \approx \frac {\epsilon D_{N}}{\Lambda }\left (1 - \frac {\hat {\mathcal {D}}}{D_{N}} \right)$. We conclude the new diffusion dynamics are on the order of

$$\begin{array}{@{}rcl@{}} u_{t} &=& \left(\hat{\mathcal{D}} + \Delta \hat{\mathcal{D}} \right) u_{xx} \approx \left(\hat{\mathcal{D}} + \epsilon \frac{D_{N}}{\Lambda} \left(1 - \frac{\hat{\mathcal{D}}}{D_{N}} \right) u_{xx} \right. \end{array} $$

This change in the solution *u*(*t*,*x*) then can then initiate further changes in the distribution of the ***M*** and ***N*** cells. A similar argument can be used for a change in the ***N*** population and it is clear if the signal generates changes in both ***M*** and ***N***, to first order we generate an altered diffusion model whose solution gives us clues as to new behavior. Without boundary conditions, the general solution to a diffusion model with diffusion constant *D* is given by

$$\begin{array}{@{}rcl@{}} \phi(t,x) &=& \frac{1}{\sqrt{4 \pi D \: t}} \: e^{-\frac{x^{2}}{4Dt}} \end{array} $$

Hence, our usual trigger solution is $u(t,x) = \frac {1}{\sqrt {4 \pi \hat {\mathcal {D}} \: t}} \: e^{-\frac {x^{2}}{4 \hat {\mathcal {D}} t}}$ which is altered to $\hat {u}(t,x) = \frac {1}{\sqrt {4 \pi \tilde {\mathcal {D}} \: t}} \: e^{-\frac {x^{2}}{4 \tilde {\mathcal {D}} t}}$.

## Results and discussion

We have been studying auto immune reactions from a theoretical point of view in this work. We begin by building a model of an auto immune response which is due to nonlinear interactions in two populations of cells ***M*** and ***N*** which are mediated by the two signals ***J*** and ***K***. We do not specify at this time what these two signals are and instead we argue from first principles. There is a third signal also, the IFN- *γ* level, which is denoted by ***I***. We let the deviations of these signals from base levels be given by ***i***, ***j*** and ***k***. Then the presence of a form of self damage in this model appears to be a consequence of the nonlinear interactions in the ***i***, ***j*** and ***k*** model:

$$\begin{array}{@{}rcl@{}} \left[\begin{array}{c} \boldsymbol{i}^{\prime}\\ \boldsymbol{j}^{\prime}\\ \boldsymbol{k}^{\prime} \end{array}\right] \approx \left[\begin{array}{ccc} G_{1\boldsymbol{i}}^{\boldsymbol{V_{0}}} & G_{1\boldsymbol{j}}^{\boldsymbol{V_{0}}} & G_{1\boldsymbol{k}}^{\boldsymbol{V_{0}}} \\ G_{2\boldsymbol{i}}^{\boldsymbol{V_{0}}} & G_{2\boldsymbol{j}}^{\boldsymbol{V_{0}}} & G_{2\boldsymbol{k}}^{\boldsymbol{V_{0}}} \\ G_{3\boldsymbol{i}}^{\boldsymbol{V_{0}}} & G_{3\boldsymbol{j}}^{\boldsymbol{V_{0}}} & G_{3\boldsymbol{k}}^{\boldsymbol{V_{0}}} \end{array}\right] \left[\begin{array}{c} \boldsymbol{i}\\ \boldsymbol{j}\\ \boldsymbol{k} \end{array}\right] \end{array} $$

where Note, if the two populations ***M*** and ***N*** coincide, this model reduces to a two dimensional model, we drop one signal, say ***k***, and we have

$$\begin{array}{@{}rcl@{}} \left[\begin{array}{c} \boldsymbol{i}^{\prime}\\ \boldsymbol{k}^{\prime} \end{array}\right] \approx \left[\begin{array}{ccc} G_{1\boldsymbol{i}}^{\boldsymbol{V_{0}}} & G_{1\boldsymbol{k}}^{\boldsymbol{V_{0}}} \\ G_{3\boldsymbol{i}}^{\boldsymbol{V_{0}}} & G_{3\boldsymbol{k}}^{\boldsymbol{V_{0}}} \end{array}\right] \left[\begin{array}{c} \boldsymbol{i}\\ \boldsymbol{k} \end{array}\right] \end{array} $$

and the chance of oscillation between the cellular population groups is lost. Hence, we can note some consequences and predictions due to our model.

- The crucial assumption here is that the triggering event has an effect on the host that divides into two parts. For a WNV infection, these two cell populations are the dividing and nondividing infected cells, ***D*** and ***N***, respectively. But here, we have posited that these two cell populations are given by ***M*** and ***N*** instead. Hence, any infectious agents or trigger that gives rise to such a *split* response engenders a similar collateral damage response which we interpret as an autoimmune reaction. We note this give us a significant insight into general autoimmune responses. Note that Fig. 1 shows there is collateral damage that oscillates due to the trigger and we could interpret a downswing in collateral damage as a *relapse* event.
- We have assumed $G_{2\boldsymbol {j}}^{\boldsymbol {V_{0}}} = +$, $G_{3\boldsymbol {j}}^{\boldsymbol {V_{0}}} = +$, $G_{2\boldsymbol {k}}^{\boldsymbol {V_{0}}} = -$, $G_{3\boldsymbol {k}}^{\boldsymbol {V_{0}}} = +$, which then says the coefficient matrix of the linearized upregulation and free antigen model has the form
  $$\begin{array}{@{}rcl@{}} \left[\begin{array}{cc} G_{2\boldsymbol{j}}^{\boldsymbol{V_{0}}} & -G_{2\boldsymbol{k}}^{\boldsymbol{V_{0}}}\\ G_{3\boldsymbol{j}}^{\boldsymbol{V_{0}}} & G_{3\boldsymbol{k}}^{\boldsymbol{V_{0}}} \end{array}\right] &=& \left[\begin{array}{cc} + & -\\ + & + \end{array}\right] \end{array} $$
  This algebraic sign pattern itself can give rise to complex eigenvalues for the linearized nonlinear interaction model and we have not explored this more general problem. Here, we have posited **specific** relations that give rise to clearcut oscillations. We have assumed $G_{2\boldsymbol {j}}^{\boldsymbol {V_{0}}} = G_{3\boldsymbol {k}}^{\boldsymbol {V_{0}}} = \alpha ^{\boldsymbol {V_{0}}}$ and $G_{3\boldsymbol {j}}^{\boldsymbol {V_{0}}} = -G_{2\boldsymbol {k}}^{\boldsymbol {V_{0}}} = \beta ^{\boldsymbol {V_{0}}}$ which gives rise to the characteristic coefficient matrix
  $$\begin{array}{@{}rcl@{}} \left[\begin{array}{cc} G_{2\boldsymbol{j}}^{\boldsymbol{V_{0}}} & -G_{3\boldsymbol{j}}^{\boldsymbol{V_{0}}}\\ G_{3\boldsymbol{j}}^{\boldsymbol{V_{0}}} & G_{2\boldsymbol{J}}^{\boldsymbol{V_{0}}} \end{array}\right] &=& \left[\begin{array}{cc} \alpha^{\boldsymbol{V_{0}}} & -\beta^{\boldsymbol{V_{0}}}\\ \beta^{\boldsymbol{V_{0}}} & \alpha^{\boldsymbol{V_{0}}} \end{array}\right] \end{array} $$

The remaining questions are then to try to understand the algebraic sign patterns from a low level analysis of the trigger signal that initiates the potential auto immune reaction. We have provided in this paper, three different ways to look at the trigger response.

- We analyze a general trigger in Section “General trigger models” and show that we can understand how the signal generates protein transcription changes via equations such as
  $$\begin{array}{@{}rcl@{}} \delta_{T_{M}} &=& \mu_{M} \: \left(2 \epsilon_{M}\: + \: \epsilon_{M}^{2}\right) \end{array} $$
  which shows how changes in the trigger generate changes in another signal via a cascade of protein transcriptions culminating in a change in fragility for ***M***.
- Then, in Section “Computational abstractions”, a computational approach using sigmoid activation function *h*_***M***_ further found that if we associate the change in fragility in ***M*** due to *T*_*M*_ to be the alteration of a protein complex *P*(*T*_*M*_), then the maximum *P*(*T*_*M*_) expression is on the order of $\Delta \: \boldsymbol {P(T_{M})}^{max} \: = \: h_{\boldsymbol {P(T_{M})}}([P(T_{M})])$ where *h* is a traditional sigmoid response function which switches the protein from a low to a high level.
- We can also model the trigger signal in terms of a diffusion process as we did in Section “Approximations to trigger modification”. The trigger effects ***M*** and ***N*** is complicated ways and in this section, we study what happens if the pathways the trigger operates on generate a change in ***M*** itself. We show this in turn alters the diffusion coefficient that controls the trigger dynamics thereby potentially altering all of the trigger pathways.

Let’s use these ideas to develop an understanding of what the oscillation conditions means at the micro level. To make the analysis accessible, let’s assume the proteins *T*_*M*_ and the protein *P*(*T*_*M*_) are the same and the same is true for *T*_*N*_ and *P*(*T*_*N*_). Then, we can write at equilibrium

$$\begin{array}{@{}rcl@{}}\delta_{T_{M}} &=& \mu_{M} \: (2 \epsilon_{M}\: + \: \epsilon_{M}^{2})\\ \Delta \: \boldsymbol{T_{M}}^{max} &=& \frac{1}{2} \hat{\mu}_{M} \: \delta_{T_{M}} \: T_{M}^{max} \: \left(1 + \tanh(\frac{\left[T_{M}\right]}{g_{M}} \right)\\ &\leq& \hat{\mu}_{M} \: \delta_{T_{M}} \: T_{M}^{max} = \hat{\mu}_{M} \: \mu_{M} \: \left(2 \epsilon_{M}\: + \: \epsilon_{M}^{2}\right) T_{M}^{max} \end{array} $$

Let $\theta _{M} = \hat {\mu }_{M} \: \mu _{M} \: T_{M}^{max}$. Note *θ*_*M*_ can be positive or negative. A similar argument can be made for *T*_*N*_. We conclude

$$\begin{array}{@{}rcl@{}} \Delta \: \boldsymbol{T_{M}}^{max} &\approx& \theta_{M} \left(2 \epsilon_{M}\: + \: \epsilon_{M}^{2}\right), \\ \Delta \: \boldsymbol{T_{N}}^{max} &\approx& \theta_{N} \left(2 \epsilon_{N}\: + \: \epsilon_{N}^{2}\right) \end{array} $$

Now ***J*** ^′^=*G*_2_(***i***,***j***,***k***) which we approximate by $\frac {\delta j}{\delta t} \approx G_{2\boldsymbol {j}}^{\boldsymbol {V_{0}}} \boldsymbol {j} + G_{2\boldsymbol {k}}^{\boldsymbol {V_{0}}} \boldsymbol {k}$. Hence over one time unit, we have $\delta j \approx G_{2\boldsymbol {j}}^{\boldsymbol {V_{0}}} \boldsymbol {j} + G_{2\boldsymbol {k}}^{\boldsymbol {V_{0}}} \boldsymbol {k}$. A similar argument shows $\delta k \approx G_{3\boldsymbol {j}}^{\boldsymbol {V_{0}}} \boldsymbol {j} + G_{3\boldsymbol {k}}^{\boldsymbol {V_{0}}} \boldsymbol {k}$. If the protein *T*_*M*_ and *P*(*T*_*M*_) are actually ***j*** and the proteins *T*_*N*_ and *P*(*T*_*N*_) are ***k***, we have

$$\begin{array}{@{}rcl@{}} G_{2\boldsymbol{j}}^{\boldsymbol{V_{0}}} &\approx& \frac{\delta \boldsymbol{j}}{ \boldsymbol{j}}\ \text{for fixed} \,\boldsymbol{k}, \quad \quad G_{2\boldsymbol{k}}^{\boldsymbol{V_{0}}} \approx \frac{\delta \boldsymbol{j} }{\boldsymbol{k}} \text{for fixed}\, \boldsymbol{j}\\ G_{3\boldsymbol{j}}^{\boldsymbol{V_{0}}} &\approx& \frac{\delta \boldsymbol{k}}{\boldsymbol{j}}\ \text{for fixed}\, \boldsymbol{k}, \quad \quad G_{3\boldsymbol{k}}^{\boldsymbol{V_{0}}} \approx \frac{\delta \boldsymbol{k}}{\boldsymbol{k}} \text{for fixed} \,\boldsymbol{j} \end{array} $$

We assume

$$\begin{array}{@{}rcl@{}} G_{2\boldsymbol{j}}^{\boldsymbol{V_{0}}} &\approx& \frac{\delta \boldsymbol{j}}{ \boldsymbol{j}} = \theta_{2j} \left(2 \epsilon_{2j} \: + \: \epsilon_{2j}^{2}\right), \quad G_{2\boldsymbol{k}}^{\boldsymbol{V_{0}}} \approx \frac{\delta \boldsymbol{j} }{\boldsymbol{k}} = \theta_{2k} \left(2 \epsilon_{2k} \: + \: \epsilon_{2k}^{2}\right)\\ G_{3\boldsymbol{j}}^{\boldsymbol{V_{0}}} &\approx& \frac{\delta \boldsymbol{k}}{\boldsymbol{j}} = \theta_{3j} \left(2 \epsilon_{3j} \: + \: \epsilon_{3j}^{2}\right), \quad G_{3\boldsymbol{k}}^{\boldsymbol{V_{0}}} \approx \frac{\delta \boldsymbol{k}}{\boldsymbol{k}} = \theta_{3k} \left(2 \epsilon_{3k} \: + \: \epsilon_{3k}^{2}\right) \end{array} $$

The oscillation conditions then imply

$$\begin{array}{@{}rcl@{}} \theta_{2j} \left(2 \epsilon_{2j} \: + \: \epsilon_{2j}^{2}\right) &=& \theta_{3k} \left(2 \epsilon_{3k} \: + \: \epsilon_{3k}^{2}\right), \quad \text{and}\\ \theta_{3j} \left(2 \epsilon_{3j} \: + \: \epsilon_{3j}^{2}\right) &=& - \theta_{2k} \left(2 \epsilon_{2k} \: + \: \epsilon_{2k}^{2}\right) \end{array} $$

from which can conclude we probably have oscillations if the algebraic sign of *θ*_3*j*_ is opposite to *θ*_2*k*_ and if the algebraic signs of *θ*_2*j*_ and *θ*_3*k*_ match. We also want *θ*_2*j*_ positive so we get undamped oscillations. The more exact equality conditions are probably not actually needed although the analysis was easier when we made them.

How do we use this information? Once we identify signals ***j*** and ***k*** useful for the dynamical model of interest, we need to experimentally estimate $\frac {\delta \boldsymbol {j}}{ \boldsymbol {j}}$, $\frac {\delta \boldsymbol {j} }{\boldsymbol {k}}, \frac {\delta \boldsymbol {k}}{\boldsymbol {j}}$ and $\frac {\delta \boldsymbol {k}}{\boldsymbol {k}}$. This will give us estimates for the algebraic signs. We believe there will be an autoimmune interaction is the sign patterns we have discussed hold.

## Conclusion

In conclusion, we have shown that we can build a reasonable model of how a trigger agent such as a virus, a bacteria or an environmental toxin can infect a host’s cell and cause an autoimmune reaction. Part of our model is a *macro* one and we believe it provides clues as to how general auto immune reactions behave. We have posited that for an infectious agent or trigger to cause oscillations in health it is required that the trigger causes alterations in two distinct cell populations. Then, if the nonlinear interactions between these two populations satisfies the conditions for damped oscillatory response we have mentioned here, we should see oscillations in the host health and collateral damage. Another part of our model is a detailed *micro* level one which looks at the triggering pathway and examines how the events in that pathway can contribute to the nonlinear interaction assumptions we make. We also discuss in detail how a sudden trigger increase can be modeled in terms of a diffusion based response and how that also can influence the nonlinear interactions we need for an auto immune response.

**Fig. 7:**
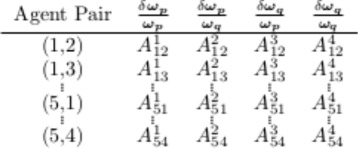
Identifying agents for the auto immune model

We will finish with a few speculations about how to decide if there is an auto immune response. If one is suspected, we could run the following experiment. Let’s assume there are *N* possible signals ***ω***_***1***_,…,***ω***_***N***_ that we think could be important in the analysis of the chosen potential auto immune response. There are many possible cytokines, chemokines and other molecular agents that could be of interest. We then setup a standard *M*×*M* well type genomic assay experiment. Each well is designed by growing a three dimensional *organoid* which will play the role of a *host*. This is quite possible and the paper [10] provides clear guidelines as to how to do this for mini human brains grown from stem cells. We would first measure cytokine and chemokine etc. levels in all of the wells as well as other expressed proteins for a given trigger level. At this stage, we are trying to find to the populations ***M*** and ***N*** which handle the trigger level differently as this is part of our assumptions that are needed to cause an auto immune response. If two such populations are discovered, then we need to identify the agents ***J*** and ***K*** to use in the model. Assume *N*=5 for convenience. Then, for each pair *p*≠*q*, we must measure the equivalents of $\frac {\delta \boldsymbol {j}}{\boldsymbol {j}}, \frac {\delta \boldsymbol {j}}{\boldsymbol {k}}$, $\frac {\delta \boldsymbol {k}}{\boldsymbol {j}}$ and $\frac {\delta \boldsymbol {k}}{\boldsymbol {k}}$. For a given *p* and *q* pair, let these measured values be $A_{pq}^{1}, A_{pq}^{2}, A_{pq}^{3}$ and $A_{pq}^{4}$. We then have the possibilities shown in Fig. 7.

We sift through Fig. 7 looking for the following: we want $A_{pq}^{1} > 0$ for undamped oscillations and we want the oscillation conditions: the sign of $A_{pq}^{3}$ is the opposite of $A_{pq}^{2}$ and the signs of $A_{pq}^{1}$ and $A_{pq}^{4}$ match. If we can find such a pair (*p*,*q*) then we have identified two signals for the two populations of ***M*** and ***N*** type cells that could imply an auto immune response is possible for this trigger. We think this is quite interesting and could help us decide if a medical event should be classified as an auto immune event.

## Methods

We consider this work essentially a theoretical model and we hope that we can generate some insights into the many troubling auto immune problems we face.
